# The Use of Force Plate Technology to Measure Force Production Characteristics in Military Personnel: A Scoping Review of Methodological Reporting Practices

**DOI:** 10.1186/s40798-025-00942-6

**Published:** 2025-11-21

**Authors:** Peter Ladlow, Kieran M. Lunt, Paul Comfort, Oliver O’Sullivan, Francisco J Robles-Palazón, Nicholas J. Ripley, John J. McMahon, Natalie Masters, Robyn P. Cassidy, Marina De Vecchis, Vanessa Bell, Alexander N. Bennett, Russell J. Coppack

**Affiliations:** 1 Academic Department of Military Rehabilitation, Defence Medical Rehabilitation Centre (DMRC) Stanford Hall, Loughborough, UK; 2 Department of Health, University of Bath, Bath, BA2 7AY UK; 3 School of Health and Society, University of Salford, Salford, UK; 4 School of Medical and Health Sciences, Edith Cowan University, Joondalup, WA Australia; 5 Academic Unit of Injury, Recovery and Inflammation Sciences, Faculty of Medicine and Health Sciences, University of Nottingham, Nottingham, UK; 6 Department of Physical Activity and Sport, Faculty of Sport Sciences, Campus of Excellence Mare Nostrum, University of Murcia, Murcia, Spain; 7 Hawkin Dynamics, Inc, Westbrook, ME US; 8 Defence Medical Academy, Defence Medical Services Whittington, Lichfield, UK; 9 Department of Bioengineering, Imperial College London, London, UK

**Keywords:** Force plates, Strength testing, Military, Performance, Injury profiling, Rehabilitation

## Abstract

**Background:**

Force plates have become one of the most frequently used assessment tools in the field of strength and conditioning research and applied practice. Their use for measuring force production characteristics among military personnel is growing. The aim of this scoping review is to: (1) Describe the current evidence base underpinning the use of force plates to assess maximal and rapid lower-limb force production in military in three priority areas; occupational task performance, injury risk profiling, and rehabilitation, (2) Identify potential trends and/or differences by participants’ sex, job role, and/or level of performance in tests, methodologies, and metrics selected, and (3) Consider gaps in the existing evidence base and questions that should be addressed in future research.

**Main Body:**

Nine hundred and eighty-five articles were identified across EBSCO and Ovid database platforms. After removing duplicates and applying the eligibility criteria, 40 articles were included in this review. Major differences/inconsistences in the methodological reporting of force plate ‘preparation’ (e.g., testing surface/flooring conditions, zeroing force plates and weighing participants during trials), ‘execution’ (e.g., verbal cueing and footwear conditions) and ‘analysis’ (e.g., software used to analyse force–time data, filters applied, thresholds used, metric selection and reliability testing) were found. The isometric midthigh pull (IMTP) and countermovement jump (CMJ) were the most used isometric and dynamic tests used, respectively. However, military researchers are commonly using no more than two metrics from each of these tests, with an emphasis towards only reporting outcome-based metrics (e.g., jump height) as opposed to movement strategy-based metrics. A more thorough review of the methodological reporting practices of the IMTP revealed most military researchers are using incorrect coaching instructions compared to standardised methodological guidelines or no instructions at all. When different expressions of force or power are reported using the CMJ, greater than half do not identify the phase of the jump it was calculated from.

**Conclusions:**

Across military populations, the methodological reporting standards and metrics selected when using force plate technology to inform ‘occupational task performance’, ‘injury profiling’ and ‘rehabilitation’ have been sub-optimal and sometimes incomplete. Force plate technology is currently under exploited when applied to military population research.

**Supplementary Information:**

The online version contains supplementary material available at 10.1186/s40798-025-00942-6.

## Key Points

1. The use of force plate technology to measure maximal and/or rapid force production characteristics in military personnel is growing rapidly.
2. The methodological reporting practices of force plate technology within military research practice is inconsistent and often poorly described.
3. If military nations are to effectively use force plate technology in the future, greater awareness of metric selection and factors that influence the quality/accuracy of data outputs is warranted.

**Supplementary Information:**

The online version contains supplementary material available at 10.1186/s40798-025-00942-6.

## Introduction

Military organisations must invest effort in physically preparing personnel for the rigours of military service while preserving the fitness and health of the workforce throughout military careers [1]. All components of fitness are required to perform physically arduous military-specific tasks successfully. However, the importance of muscular strength has been recognised in military environments, where occupational tasks require repeated load carriage, loaded marching, and manual handling, such as lifting, digging, carrying, pushing, pulling and their combinations, as well as casualty evacuation [2–4]. In addition, the ability to produce high forces (maximum strength) provides the foundations of a soldier’s ability to create explosive movements necessary during close-quarter combat, jumping/landing, multidirectional speed and agility, sprinting and throwing [5]. Furthermore, strength training has been consistently shown to be a vital component of MSKI reduction strategies [6]. Therefore, improving or maintaining both maximal strength and the ability to apply force rapidly (i.e., rate of force development [RFD]) is essential to optimise the number of military personnel fit for operational duty [7]. Rapid force production is important as it is the force relative to the mass being moved that determines acceleration, with the duration over which the force is applied that determines the resulting velocity (i.e., impulse = ∆force × ∆time), based on the impulse momentum theorem [8–10]. To meet this task, physical training staff and rehabilitation practitioners must tailor their programmes to meet individual’s functional needs and the occupational demands of their unit. Therefore, valid, reliable and sensitive measures of physical performance assessment, including maximal and rapid force production characteristics are essential.

Physical fitness testing commonly features as a part of military recruitment, selection courses, regular follow-up of individual and unit readiness and guiding return to full deployability status following MSKI. The availability of objective measures of functional strength is required to allow physical training staff and rehabilitation practitioners to track physical fitness levels, enforce physical fitness standards, profile injury risk predisposition, modulate the rehabilitation process over time, determine the effectiveness of training regimens, and identify individual unit strengths and weaknesses [7, 11].

Due to logistical challenges (e.g., equipment availability and time constraints), the ability to measure maximal and rapid force production in military populations is often constrained by the trade-off between test validity and field expediency [12]. Traditional tests of maximum strength (i.e., one-repetition maximum) employed in tactical settings tend to be more technical (i.e., requiring a high skill level), posing a potential greater risk of injury [13]. A fundamental limitation of field tests measuring ‘*explosive strength*’, for example, the vertical jump test (often conducted using a tape measure, Vertec equipment or a jump mat), is that these methods restrict practitioners to a single outcome measure, jump height. Although this metric can be useful, it fails to provide more valuable information on movement strategies, which may be more sensitive to training loads [14]. For example, whether the individual requires improvements in force or velocity capabilities (or both) to maximise their structured training methods [15]. Therefore, measuring force–time characteristics (via force plate technology) may facilitate a more comprehensive evaluation of neuromuscular performance than recording the physical outcome (e.g., jump height) alone [16]. It is also feasible that jump height may remain constant, but the time to take-off (TTT) could change, with a concomitant increase in TTT indicating fatigue or a concomitant decrease in TTT indicating an enhanced rapid force production capability [17, 18]. Any change in strategy (e.g., jump phase duration and impulse) is important to determine as it is not strength that determines jump height, but relative net propulsive impulse [8–10]. As such change in mean propulsive force, propulsive phase duration or body mass can notably affect relative net propulsive impulse and therefore jump height.

The UK military and Defence Medical Services (DMS) consider objective and subjective ‘outcome measures’ and ‘identifying and reducing MSKI’ risk as examples of leading research priorities [19, 20]. The most common reason for a medical non-deployable status among military personnel is musculoskeletal injury (MSKI) [21–24]. Armed Forces personnel injured during basic training, field exercise, and sport may be unable to deploy on operations, while soldiers injured during deployment may not be fit to return to active duty [25], thereby compromising operational readiness by reducing their operational strength [26]. Subsequently, there is a large economic and operational cost within Defence associated with MSKI. As an example, in 2007 MSKI resulted in 2.4 million annual healthcare visits and 25 million limited duty days across all service branches of the US military and more than $548 million in annual direct patient care costs across the Department of Defence [27].

Efforts to advance human performance should constantly evolve and utilise emerging science and technology capabilities to address ever-changing environmental and operational specific demands [28]. Portable force plates have become one of the most frequently used assessment tools in the field of strength and conditioning research and applied practice [29, 30], specifically for occupational task performance profiling, neuromuscular fatigue monitoring and guiding return from injury [31]. Commercially available force plate systems have been validated against industry gold standard systems [32–34], and well-established criterion data analysis procedures [35, 36]. However, not all software packages adhere to these data analysis procedures, making some comparisons between systems problematic [35, 36]. For example, there are two recommended thresholds for the identification of the onset of movement recorded immediately prior the commencement of the countermovement jump (CMJ). They include recording 30 ms before the onset of movement, and a decrease in force which exceeds 5 standard deviations of the force measured during a 1 s period of quiet standing [37], However, this is not the default threshold of some software systems [35, 36]. Such differences in onset thresholds can affect all temporal variables and even the calculation of velocity and as such when comparing data to published data, careful consideration of not only the testing methodologies, but also the data analysis procedures are essential. Due to almost instantaneous, detailed analysis and inspection of force–time characteristics combined with increased availability and affordability, the use of this technology is becoming increasingly widespread across military settings [14]. During large scale data collection events, automated analyses are preferable as it facilitates more efficient handling of force–time data, reduces human error and offers real-time feedback and results that would otherwise be unavailable to the physical training staff [35]. Indeed, the US Department of Defence have invested heavily in force plate technologies, with all four major branches of the US military now using this technology in their training and operational settings to increase the health and readiness of US military personnel [38]. An example of how the US military is using force plate technology includes evaluating physical training demands and characteristics associated with performance outcomes during the 13 week US Marine recruit training programme [39].

Due to the growing volume of published literature, there is now heightened awareness and interest in the application of force plate technology to inform physical preparation strategies, MSKI mitigation strategies and guiding rehabilitation practices across military settings [7]. However, it is important to ensure the testing procedures in place are reliable and trustworthy to inform future policy and practices. The application of force plate testing presents practitioners with a multitude of software and testing application options, with large amounts of data and force–time metrics to choose from [14]. For example, a CMJ (considered one of the simplest jump tests to administer) has more than one hundred different variables that can be obtained immediately via commercial force plate software [40]. This can often leave practitioners quickly inundated with large quantities of data that is poorly understood and often improperly actioned compared to common singular outputs (i.e., jump height recorded in m) [16, 41]. It is worth noting that some of these metrics are duplicates (e.g., TTT, movement time, contraction time) and that many others could be eliminated from use with appropriate biomechanical knowledge (e.g., rate of velocity development, which should be termed acceleration).

It is unclear what standard of methodological reporting and practices are currently being administered across military settings. The quality of data returned from force plate software relies on solid preparation of the testing protocols, coaching and cueing to confirm maximal efforts, and selection of reliable and practical metrics that can be used [16]. Selecting the correct tests and force–time metrics is crucial. However, it is also unclear which tests and metrics are currently being used across military settings and whether applied researchers and practitioners are appropriately and maximally exploiting the data that can be harnessed from force plate technology to provide betterment to the end user (i.e., military personnel, senior leadership and command staff).

This scoping review aims to identify existing methodological reporting practices used by military organisations to measure force plate derived measures of lower-limb maximal and rapid force production. The aspiration is that this might improve practitioner decision-making around the use of force plate tests and variable selection in relation to occupational task performance, injury profiling and rehabilitation practices of military personnel. The aims of this scoping review are to:

1. Describe the current evidence base underpinning the use of force plates to assess maximal and rapid lower-limb force production in military in three priority areas of occupational task performance, injury risk profiling and rehabilitation.
2. Identify potential trends and/or differences by participants’ sex, job role, and/or level of performance in tests, methodologies, and metrics selected, and
3. Consider key gaps in the existing evidence base and new questions that should be addressed in future research.

## Methods

The framework proposed by the Joanna Briggs Institute (JBI) was followed to conduct this scoping review [42]. The Preferred Reporting Items for Systematic Reviews and Meta-Analyses specific extension for Scoping Reviews (PRISMA-ScR) [43] was used to guide the reporting of the scoping review findings (Supplementary File 1). As proposed by the JBI framework, the inclusion criteria followed the elements of population, concept, and context (PCC). An overview of the PCC analysis and rationale for the eligibility criteria, alongside the literature search terms/criteria can be found in Supplementary File 2. For a summary of the aims and conclusions for each article included in this review, see Supplementary File 3.

### Eligibility Criteria

#### Participants

The participants of interest for this review were military personnel only. No limitation by participants’ age, sex, and/or level of performance was applied. This included recruits/cadets, those in regular/productive service and reservists/conscripts. Any article that included civilians among its primary cohort was excluded. Veterans were also excluded.

#### Concept

Force plate methodologies applied to assess maximal and rapid lower-limb force production for military occupational task performance, injury profiling or during rehabilitation were reviewed. For avoidance of doubt, if force plates were used during a rebound task (e.g., such as a drop vertical jump [DVJ]) but reported landing-based metrics only (i.e., no propulsion force time characteristics), such articles were excluded. Articles that used force plates to assess balance and stability only or those assessing reliability and/or validity of testing procedures only were excluded.

For this review, an injury was defined as any musculoskeletal damage, involving bones, muscles, ligaments, tendons, joints and associated tissues. No exclusions were made based on the type of injury (e.g., primary and secondary injuries). Metrics derived from the combination of data obtained through different technologies (e.g., joint moments) were beyond the scope of this review.

#### Context

The context of this scoping review was any setting where military personnel undergo physical assessments to evaluate their health and/or occupational task performance status.

### Sources

Only articles with a quantitative study design, published in a peer-reviewed journal were retained. Literature reviews, conference abstracts, editorial commentaries, pre-prints, and letters to the editor were excluded to avoid duplication of data.

### Search Strategy

**Fig. 1:**
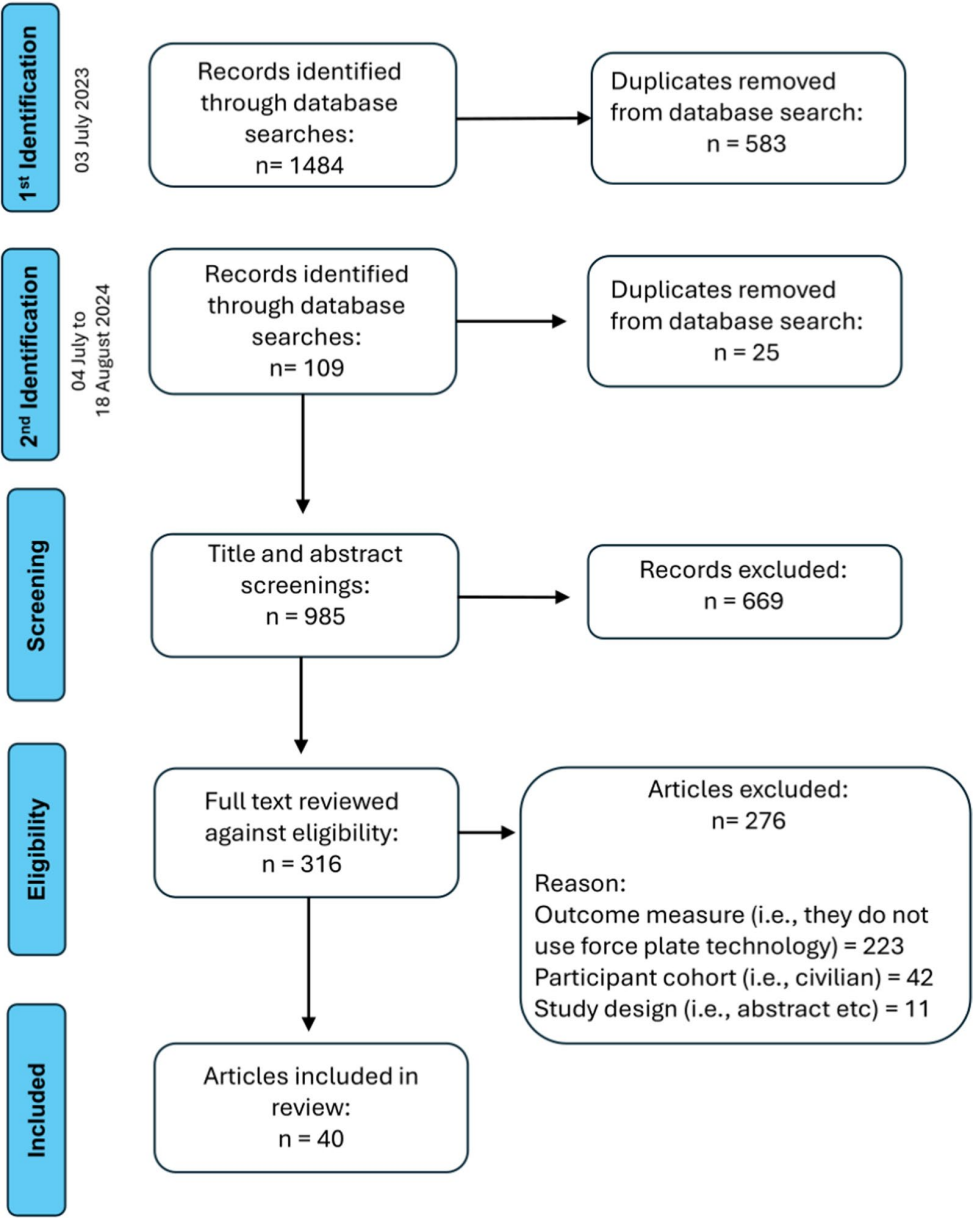
Article Selection process

A systematic search was conducted by a research librarian (VB) up to 18 August 2024 in the databases MEDLINE/SPORTDiscus and CINAHL using the EBSCO platform and EMBASE using the Ovid platform. Relevant search terms were used to construct Boolean search strategies in the different databases. Examples of the search strategy applied are found in Supplementary File 2. Only studies published in English were considered for inclusion in this review. No date restrictions were applied. Articles were stored using Covidence online systematic review software (Covidence.org) for screening. Duplicates were identified within Covidence and removed. The screening process was conducted by two authors (PL and KL) and was blinded. First, studies were screened based on title and abstract; second, the full text retained after initial screening was reviewed against the eligibility. After each screening phase, any discrepancies between authors (PL and KL) were resolved by a third author (OOS). A diagrammatic description of the screening process before arriving at the final group of articles selected for review is provided in Fig. 1.

### Extraction of Results

A codebook was designed to standardise data extraction. Subsequently, each article included in this review was codified by two different authors (PL and KL). The moderator variables of the eligible studies were inspired by Robles-Palazon et al. [44] and grouped into 3 categories: (1) general study descriptors (e.g., study design, year of publication, context, injury type/s); (2) study population (e.g., number of participants, age, sex, level of performance); and (3) force plate assessment characteristics. Force plate assessment characteristics reviewed included test, instructions, number or time of registered trials, rest between trials, and metrics used. Other force plate characteristics, such as brand, model, sampling rate, filters, or software, were also recorded.

### Quality Assessment and Risk of Bias

The methodological quality of the studies was assessed by analysing the reproducibility of the methodologies based on force plate variables that align with 3 themes: methodological ‘preparation’, ‘execution’ and ‘analysis’ of force plate administration. The number/count of criteria met (under each theme listed above) was used as a proxy for the ‘quality’ of methodological reporting, with higher counts indicating higher quality. Risk of bias assessment was performed using the Newcastle–Ottawa Scale (NOS) assessment tool. Three reviewers (PL, KL and OOS) ensured each article was independently reviewed on two occasions [45].

### Analysis of Data

Only descriptive data is provided in this review and presented as number, percentages, means and standard deviations.

## Results

To facilitate interpretation, results are described as follows: Table 1 summarises the demographics of each article that met the eligibility criteria and Table 2 details the force plate specifications and analysis used. For tests measuring either ‘isometric’ or ‘dynamic’ force production, methodological reporting was reviewed across three themes (preparation, execution and analysis), accompanied by a risk of bias assessment. Supplementary file 3 summarises the article aims and conclusions. Finally, a more comprehensive review of methodological reporting practices of the most used force plate tests (isometric mid-thigh pull [IMTP] and CMJ) identified specific information regarding test technique, verbal cueing, test repetitions, rest duration, duration of test (specific to isometric tasks), force plate metrics used, and summary of results. Tables 3 and 4 describe the above structure of results aligned to ‘isometric’ tasks and the IMTP, respectively. Tables 5 and 6 describe the above results aligned to ‘dynamic’ tasks and the CMJ, respectively. Force plate derived strength data for the IMTP and CMJ tests are presented in supplementary file 4 Tables S4 and S5, respectively.

**Table 1:** Article information: author, year published, military organisation, personnel demographics, study design, type and test undertaken

Study	Year	Military nation	Military branch	Career status	Participants at baseline (*n*)	Males *(n)*	Females (*n*)	Age (m ± SD [years])	Study design	Type of study	Test(s) used
Angelviet et al. [61]	2016	Norway	Navy	Regulars	21	–	–	28 ± 4	Cross–sectional	Performance	CMJ
Barrett et al. [47]	2024	US	Marines	Regulars	112	–	–	22 ± 3	Cohort	Performance	CMJ
Bird et al. [48]	2022	US	Marines	Officer Cadets	728	599	129	25 ± 3	Cohort	Injury Profiling	CMJ
Bird et al. [49]	2023	US	Marines	Officer Cadets	689	566	123	25 ± 3	Cohort	Injury Profiling	CMJ
Burley et al. [67]	2020	Australia	Army	Recruits	214	162	52	Exp 22 ± 4, Con 21 ± 4	RCT	Performance	SJ
Chassé et al. [77]	2019	Canada	Army	Regulars	17	9	8	34 ± 8	Cross sectional	Performance	IMTP
Conkright et al. [50]	2021	US	mixed	Reservists	69	54	15	M 26 ± 5 F 26 ± 6	Non-RCT	Performance	CMJ
Debenedictis et al. [68]	2021	Australia	Army	Regulars	16	16	0	22 ± 2	RCT	Performance	Drop Jump
Doyle et al. [69]	2022	Australia	Army	Regulars	82	82	0	27 ± 3	Prospective observational	Injury Profiling	CMJ + SJ + IMTP
Groeller et al. [70]	2015	Australia	Army	Recruits	51	45	6	21 ± 3	Prospective observational	Performance	SJ
Hamarsland et al. [62]	2018	Norway	Navy	Regulars	15	–	–	28 ± 5	Prospective observational	Performance	CMJ + LP
Hando et al. [51]	2022	US	Air Force	Recruits	823	823	0	23 ± 4	Prospective observational	Injury Profiling	CMJ
Johnson et al. [52]	2021	US	Marines	Regulars	46	46	0	Injury 29 ± 4 Con 28 ± 4	Cross sectional	Rehab/Injury Profiling	Stop Jump
Karatrantou et al. [80]	2019	Greece	Air Force	Academy Cadets	60	60	0	20 ± 1	RCT	Performance	CMJ + SJ
Kozinc et al. [72]	2021	Slovenia	Army	Regulars	97	89	8	Exp 31 ± 6 Con 34 ± 6	RCT	Performance	CMJ
Lovalekar et al. [53]	2024	US	Marines	Cadets	584	401	183	19 ± 2	Cohort	Injury Profiling	CMJ + IMTP
McFadden et al. [54]	2024a	US	Marines	Recruits	274	176	98	19 ± 2	Cohort	Performance	CMJ + IMTP
McFadden et al. [39]	2024b	US	Marines	Recruits	135	65	70	–	Cohort	Performance	CMJ + IMTP
Merrigan et al. [46]	2021	US	Marines	Regulars	34	18	16	–	Cohort	Performance	CMJ
Merrigan et al. [55]	2022	US	Army	Reservist recruits	26	14	12	21 ± 3	Cross sectional (Randomised Cross-over)	Performance	Drop Jump
Nevin et al. [75]	2024	UK	Army	Regulars	39	39	0	31 ± 6	Cross sectional	Performance	IMTP
Øfsteng et al. [63]	2020	Norway	Army	Recruits	38	31	7	22 ± 1	RCT	Performance	CMJ
Orantes-Gonzalez et al. [82]	2022	Spain	Army	Regulars	40	40	0	26 ± 5	Randomised Cross over	Performance	SJ
Peterson et al. [56]	2024	US	Marines	Recruits	565	386	179	M = 19 ± 2 F = 20 ± 2	Cohort	Performance/Injury Profiling	CMJ + IMTP
Pihlainen et al. [79]	2018	Finland	Army	Regulars	81	81	0	30 ± 8	Cross sectional (pre-post under loaded conditions)	Performance	CMJ
Poser et al. [57]	2019	US	Army	Reserves	18	18	0	24 ± 6	Cross sectional	Performance	Deadlift
Potter et al. [58]	2023	US	Marines	Regulars	736	0	736	30 ± 7	Cross sectional	Performance	CMJ
Robitaille et al. [78]	2024	Canada	Not reported	Recruits	98	95	3	22 ± 3	Cohort	Injury Profiling	IMTP
Rue et al. [76]	2023	UK	Army/RAF	Recruits	381	339	42	JE 16 ± 1 SE 21 ± 4 RAF 21 ± 3	Cohort	Performance	IMTP
Scott et al. [59]	2022	US	Air Force	Recruits	643	643	0	22 ± 4	Cohort	Performance	CMJ
Šimenko et al. [73]	2021	Slovenia	Army	Regulars	181	181	0	31 ± 6	Cross-sectional	Performance	CMJ
Smith et al. [71]	2023	Australia	Army	Regulars	17	13	4	Con, 31 ± 9 Exp, 25 ± 4	RCT	Performance	CMJ + SLCMJ
Solberg et al. [64]	2015	Norway	Navy	Regulars	22	–	–	28 ± 4	Non-randomised longitudinal study	Performance	CMJ
Thompson et al. [28]	2023	US	Marines	Regulars	34	18	16	M 26 ± 8 F 24 ± 3	Cohort	Performance	CMJ
Vikmoen et al. [65]	2020	Norway	Not reported	Conscripts	35	23	12	M 19 ± 2 F 19 ± 2	Observational	Performance	CMJ
Vikmoen et al. [66]	2024	Norway	Cyber Defence	Recruits	18	10	8	M: 21 ± 1 F 21 ± 1	Observational	Performance	CMJ
Vodičar et al. [74]	2022	Slovenia	Army	Regulars	118	118	0	30 ± 6	Cross-sectional	Performance	CMJ
Walters et al. [12]	2022	UK	Mixed	Regulars	19	12	7	34 ± 8	Cross-sectional	Rehabilitation	IMTP
Yanovich et al. [81]	2008	Israel	Army	Recruits/conscripts	176	47	129	19 ± 1	Cohort	Performance	CMJ + SLCMJ
Zifchock et al. [60]	2024	US	Marines	Regulars	111	110	1	Course 1 21 ± 2 Course 2 23 ± 4	Cohort	Performance	CMJ

SD: standard deviation; US: United States; UK: United Kingdom; CMJ: countermovement jump; SJ: Squat Jump; IMTP: isometric mid-thigh pull; SLCMJ: single leg countermovement jump; M: male; F: Female; Exp: experimental group; Con: control group; LP: Leg Press

**Table 2:** Force Plate Specifications and analysis

Study	Test used	Force plate brand and model	Sampling rate (Hz)	Software used to process/analyse data
Angelviet et al. [61]	CMJ	HUR Labs	Not reported	Excel and SPSS
Barrett et al. [47]	CMJ	Sparta Science	Not reported	Sparta Science software
Bird et al. [48]	CMJ	Hawkin Dynamics	1000	Hawkin Dynamics Software
Bird et al. [49]	CMJ	Sparta Science	1000	Sparta Science Software
Burley et al. [67]	SJ	Kistler 9260AA6	2000	BioWare
Chassé et al. [77]	IMTP	Pasco PASPORT PS-2141	Not reported	Not reported how force–time data was processed/analysed
Conkright et al. [50]	CMJ	Kistler (no model)	Not reported	BioWare and customized script in MatLab
Debenedictis et al. [68]	DVJ	Kistler 9821A	Not reported	Not reported how force–time data was processed/analysed Statistics using STATA
Doyle et al. [69]	CMJ + SJ + IMTP	Fitness Technology Series 400S + Performance	600	Custom software using MatLab
Groeller et al. [70]	SJ	Kistler 9260AA6	2000	Bioware
Hamarsland et al. [62]	CMJ + Leg Press	CMJ—HUR Labs Leg Press—Custom made machine with bar connected to force plates	Both CMJ and IMTP—Not reported	Not reported how force–time data was processed/analysed. Statistics using GraphPad
Hando et al. [51]	CMJ	Sparta Science	Not reported	Sparta Science proprietary software
Johnson et al. [52]	Stop jump	Kistler 9286BA	1200	Biomechanics data processed using Visual 3D
Karatrantou et al. [80]	CMJ + SJ	Bertec	Not reported	Not reported how force–time data was processed/analysed. Statistics using SPSS
Kozinc et al. [72]	CMJ	Kistler 9286AA	Not reported	MARS software (Kistler Instrument)
Lovalekar et al. [53]	CMJ + IMTP	VALD ForceDecks	1000	ForceDecks software + Stata
McFadden et al. [54]	CMJ + IMTP	VALD ForceDecks	1000	R Studio
McFadden et al. [39]	CMJ + IMTP	VALD ForceDecks	1000	R Studio
Merrigan et al. [46]	CMJ	VALD ForceDecks (FD4000)	1000	ForceDecks software + R Studio
Merrigan et al. [55]	Drop Jump	Bertec FP 4060-NC	1000	MATLAB
Nevin et al. [75]	IMTP	Hawkin Dynamics v3	1000	Hawkin Dynamics software
Øfsteng et al. [63]	CMJ	AMTI—SG-9	1000	Not reported how force–time data was processed/analysed Statistics using R studio
Orantes-Gonzalez et al. [82]	SJ	AMTI—OPT464508HF	1000	AMTI AccuPower 3.0 software
Peterson et al. [56]	CMJ + IMTP	VALD FDLite ForceDecks	1000	VALD ForceDecks software and R Studio
Pihlainen et al. [79]	CMJ	HUR Labs FP8	Not reported	HUR Labs Force Platform Software
Poser et al. [57]	Deadlift	AMTI AccPower	Not reported	Not reported how force–time data was processed/analysed Statistics using SPSS
Potter et al. [58]	CMJ	AMTI	Not reported	AccuPower Solutions
Robitaille et al. [78]	IMTP	Hawkin Dynamics and Vernier	1000 and 50	Not reported how force–time data was processed/analysed Statistics using SPSS
Rue et al. [76]	IMTP	Pasco PASO10660	Not reported	Not reported how force–time data was processed/analysed Statistics using SPSS
Scott et al. [59]	CMJ	Bertec/Sparta Science	1000	Sparta Science proprietary software
Šimenko et al. [73]	CMJ	Kistler 9286AA	Not reported	ARS Software (Science to Practice)
Smith et al. [71]	CMJ + SLCMJ	Bertec/Sparta Science SSFP01	1000	Sparta Science proprietary software
Solberg et al. [64]	CMJ	HUR Labs	Not reported	Not reported how force–time data was processed/analysed or the statistical software used
Thompson et al. [28]	CMJ	VALD ForceDecks FD4000	1000	R Studio
Vikmoen et al. [65]	CMJ	HUR Labs	Not reported	Not reported how force–time data was processed/analysed. Statistics using SPSS
Vikmoen et al. [66]	CMJ	HUR Labs	Not reported	Not reported how force–time data was processed/analysed. Statistics using SPSS
Vodičar et al. [74]	CMJ	Kistler 9286AA	Not reported	Not reported how force–time data was processed/analysed or the statistical software used
Walters et al. [12]	IMTP	VALD ForceDecks V2.1	1000	VALD Integrated analysing software
Yanovich et al. [81]	CMJ + SLCMJ	Orthometrix Leonardo ground reaction force plates	Not reported	Not reported how force–time data was processed/analysed or the statistical software used
Zifchock et al. [60]	CMJ	VALD ForceDecks	Not reported	MATLAB

CMJ: countermovement jump; SJ: squat jump; IMTP: isometric mid-thigh pull; DVJ: drop vertical jump; SLCMJ: single leg countermovement jump

**Table 3:**
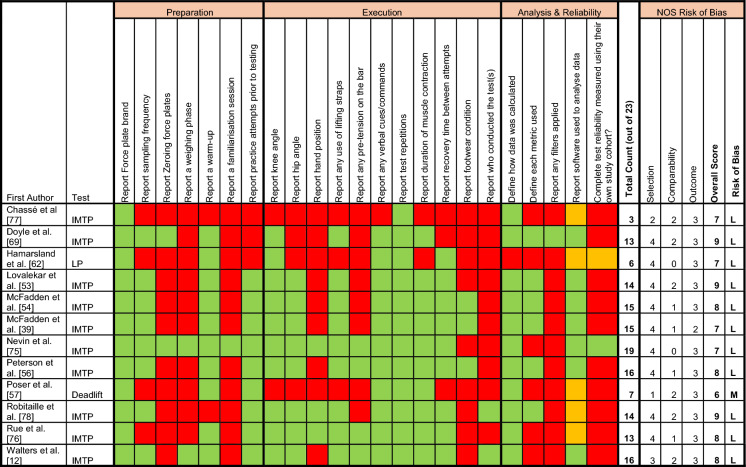
Summary of force plate related methodological reporting across all isometric tasks

IMTP: isometric mid-thigh pull; LP: leg press; L: Low; M: Moderate; Green: reported; Orange: partly reported; Red: not reported;

**Table 4:** Methodological reporting of Isometric Mid-Thigh Pull (IMTP) Test—Execution

Study	Test execution/technique	Verbal cues	Test repetitions (*n*)	Rest between attempts	Duration of Test (s)
Chassé et al. [77]	No mention of Knee or hip angle No mention of handgrip position No mention of Lifting straps being used No mention of pre-tension applied to bar	Not reported	3	Not reported	Not reported
Doyle et al. [69]	Knee angle ~ 130°. No mention of hip angle No mention of handgrip position Lifting straps were used No mention of pre-tension applied to bar	“*Pull* the bar as hard and as fast as possible”	2	Not reported	5
Lovalekar et al. [53]	Knee angle 125–145°. Hip angle 140–150° No mention of handgrip position Lifting straps were used No mention of pre-tension applied to bar	“*Pull* as hard and fast as possible”	2 (3 if > 250 N difference between trials)	2 min	5
McFadden et al. [54]	Knee angle 125–145°. Hip angle 140–150° No mention of handgrip position Weightlifting straps were used No mention of pre-tension applied to bar	“*Pull* as hard and fast as possible”	2 (3 if > 250 N difference between trials)	− 2 min	5
McFadden et al. [39]	Knee angle 125–145°. Hip angle 140–150° No mention of handgrip position Lifting straps were used No mention of pre-tension applied to bar	“*Pull* as hard and fast as possible”	2 (3 if > 250 N difference between trials)	− 2 min	5
Nevin et al. [75]	Knee angle 125–145°. Hip angle 140–150° Angles inspected using a goniometer Pronated handgrip position Lifting straps were used ‘Minimal tension was applied to bar (< 50 N)’	“*Push* their feet into the ground as fast and as hard as possible.”	3	2 min	~ 5
Peterson et al. [56]	Knee angle 125–145°. Hip angle 140–150° No mention of handgrip position Lifting straps were used ‘Minimal pre-tensioning was permitted’	“*Pull* as hard and fast as possible”	2 (3 if > 250 N difference between trials)	~ 2 min	5
Robitaille et al. [78]	Knee angle 120–130°. Hip angle 140–150° Grip on the bar was optional (pronation, supination, or pronation–supination) Lifting aides were not permitted No mention of pre-tension applied to bar	“*Pull* as hard and fast as they could”	2	30 secs	5
Rue et al. [76]	Knee angle 120–135°. Hip angle 140–150° Pronated/overhand grip on the bar Lifting straps were used Pre-tension (‘Take up the slack’ of the bar) used	“*Pull* upwards hard and fast”	2	1 min	5
Walters et al. [12]	Knee angle 125°-145°. Hip angle 140–150°. Goniometers were used to verify joint angles No mention of handgrip position Lifting straps were used ‘Small amount of pretension used prior to maximum effort’	“*Drive* their feet into the ground as hard and as fast as possible”	3	2 min	5

**Table 5:**
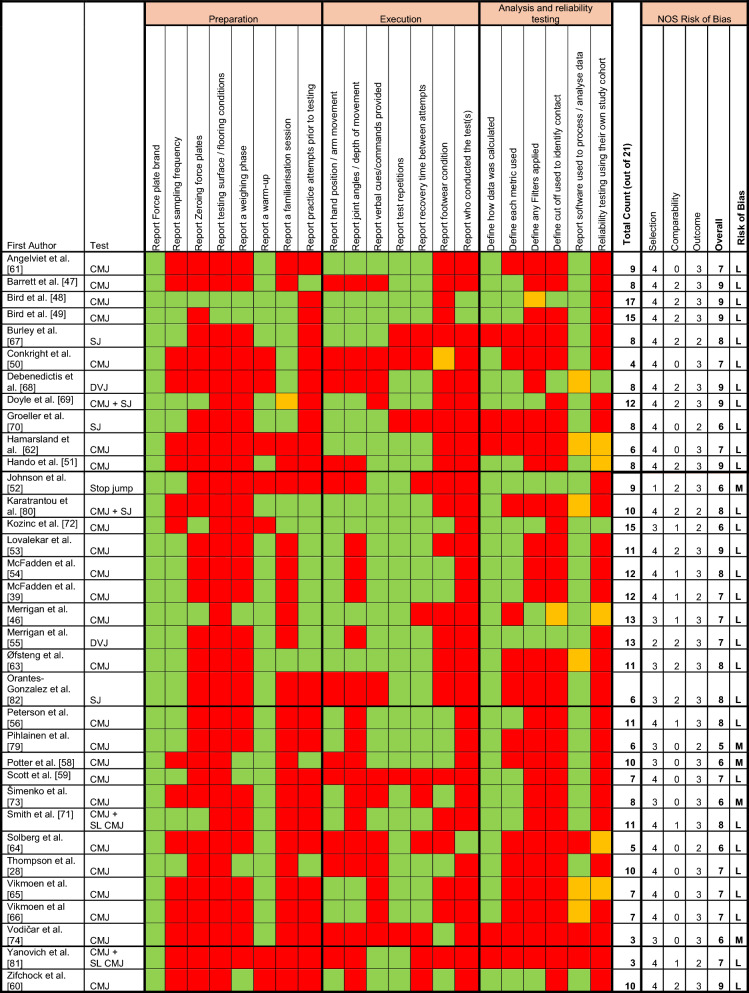
Summary of force plate related methodological reporting across all dynamic tasks

CMJ: countermovement jump; SJ: squat jump; DVJ: drop vertical jump; SL: single leg; L: Low; M: Moderate; Green: reported; Orange: partly reported; Red: not reported

**Table 6:** Methodological Reporting of Countermovement Jump (CMJ)—Execution

Study	Test execution/technique and verbal cues	Test repetitions (*n*)	Rest between repetitions (s)
Angelviet et al. [61]	Hands on hips Flex to “about 90 degrees in the knees and hips” Jump as high as possible	3	30
Barrett et al. [47]	Hand position/arm movement not reported Knee/hip angles or depth of movement not reported No verbal commands for jump initiation/execution are reported	5	10
Bird et al. [48]	Cued to start with hands above head, arm swing instructed self-selected depth Jump immediately after researchers verbally gave a 3–2-1 countdown	3	15
Bird et al. [49]	Cued to start with hands above head, arm swing instructed self-selected depth Jump immediately after researchers verbally gave a 3–2-1 countdown	3	15
Conkright et al. [50]	Hand position/arm movement not reported Joint angles/depth of movement not reported No verbal commands for jump initiation/execution are reported	Not reported	Not reported
Doyle et al. [69]	Hands on hips Flexing down to a self-selected squat depth No verbal commands for jump initiation/execution are reported	2	Variable—not timed
Hamarsland et al. [62]	Hands on hips Flex to “About 90-degree knee angle” Instructed to jump as high as possible upon reaching desired movement depth	3	30
Hando et al. [51]	Hand position/arm movement not reported Joint angles/depth of movement not reported Instructed to “jump as high as you can”	6	15
Karatrantou et al. [80]	Hands on hips Rapidly bending the knee to 90 degrees (knee angle was measured using a goniometer) Instructed to rapidly extend their knees to jump as high as possible	3	60
Kozinc et al. [72]	Hands on hips Self-selected depth Instructed to use fast countermovement to a self-selected depth, followed immediately by explosive upward propulsion to reach maximal jump height *2 conditions:* Unloaded, participants wore their boots and the uniform. Loaded, they also wore full body armour, including the helmet (total mass = 8.4 kg)	4 (2 per condition)	60
Lovalekar et al. [53]	Hands on hips Joint angles/depth of movement not reported Participants instructed to jump “as high and as fast as possible”	2	120
McFadden et al. [54]	Hands on hips Joint angles/depth of movement not reported Participants instructed to jump “as high and as fast as possible”	3	~ 120
McFadden et al. [39]	Hands on hips Joint angles/depth of movement not reported Participants instructed to jump “as high and as fast as possible”	3	~ 120
Merrigan et al. [46]	Hands grasping a pipe/bar placed across shoulders Self-selected depth Following a 3–2-1 countdown, jump “as quickly and explosively as possible” *3 conditions:* unloaded, with a polyvinyl pipe, loaded with a 10 kg tactical fitness weighted vest, and loaded with a 20kg barbell	2 for each condition	Not reported
Øfsteng et al. [63]	Hands on hips Feet were positioned hip width apart Descend to a squat position of self-selected depth and immediately jump upward Instructed to jump as high as possible	3 (4 if 3rd attempt is highest)	30
Peterson et al. [56]	Hands on hips Joint angles/depth of movement not reported Participants instructed to jump “as high and as fast as possible”	3	~ 120
Pihlainen etal. [79]	Hand position/arm movement not reported Joint angles/depth of movement not reported No verbal commands for jump initiation/execution are reported *3 conditions;* 1) unloaded 2) loaded (wearing military uniform [boots, helmet, body armour, modular vest]) and 3) loaded (military uniform + rifle)	2 unloaded, 3 for loaded condition	30
Potter et al. [58]	Hand position/arm movement not reported Joint angles/depth of movement not reported Instructed to “jump vertically for maximal height” and “land with both feet striking the platform simultaneously”	3	15
Scott et al. [59]	Hand position/arm movement not reported Joint angles/depth of movement not reported No verbal commands for jump initiation/execution are reported	Not reported	Not reported
Šimenko et al. [73]	Hands on hips Joint angles/depth of movement not reported No verbal commands for jump initiation/execution are reported *2 conditions;* 1) in uniform and boots, 2) additional body armour and a helmet (+ 8.4 kg)	2 for each condition	Not reported
Smith et al. [71]	Arm swing used Joint angles/depth of movement not reported Upon hearing an auditory cue that indicated stabilisation of body weight, Participants were instructed to perform a maximal jump height	4	15
Solberg et al. [64]	Hand position/arm movement not reported Joint angles/depth of movement not reported No verbal commands for jump initiation/execution are reported	3	Not reported
Thompson et al. [28]	Hand position/arm movement not reported Joint angles/depth of movement not reported No verbal commands for jump initiation/execution are reported *3 conditions,* 1) unloaded (holding a polyvinyl chloride pipe on the upper back), 2) loaded (wearing a 9.07 kg plate carrier with arms crossed holding the shoulder straps), and 3) (20 kg barbell positioned on the upper back)	9 (3 for each condition)	60
Vikmoen et al. [65]	Hands on hips Instructed to flex the knee and hip joint to about 90° with feet positioned shoulder-width apart Following a countdown from the test-administrator, the soldiers completed the jump	3 (4 if 3rd attempt was highest)	30
Vikmoen [et al. 66]	Hands on hips Instructed to flex the knee and hip joint to about 90° with feet positioned shoulder-width apart Following a countdown from the test-administrator, the soldiers completed the jump	3 (4 if 3rd attempt was highest)	30
Vodičar et al. [74]	Hand position/arm movement not reported Joint angles/depth of movement not reported No verbal commands for jump initiation/execution are reported *2 conditions;* 1) unloaded (wearing uniform and boots) and 2) loaded (wearing body armour and a helmet)	Not reported	Not reported
Yanovich et al. [81]	Hand position/arm movement not reported Joint angles/depth of movement not reported Instructed to “jump as high as possible”	3	Not reported
Zifchock et al. [60]	Arm swing used Joint angles/depth of movement not reported Instructed to jump as high as possible	5	Not reported

A total of 985 title and abstract screenings were screened by PL and KL, followed by full test review of 316 articles against the eligibility criteria with 40 articles included in the final review (see Fig. 1).

### Article Demographics

The 40 articles included 7,463 military personnel. Four articles did not specify participant numbers based on sex (*n* = 170 participants). Of those that did 73% were men (*n* = 5429) and 27% were women (*n* = 1864). Twelve articles (30%) did not include women. Based on the mean age reported, most personnel were < 25 years (*n* = 19), followed by 25–29 years (*n* = 11), 30–34 years (*n* = 8). No articles reported participants with a mean age greater than 35 years. Two articles did not report participant age [39, 46].

At the date of performing the final literature search (August 2024), all but one article was published within the past decade, with 33 articles (83%) published within the past 5 years and 16 articles (40%) within the previous 2 years. Most articles involved US military personnel (*n* = 17) [28, 39, 46–60], followed by Norwegians (*n* = 6) 61–66], and Australians (*n* = 5) [67–71]. Others included Slovenia (*n* = 3) [72–74], UK (*n* = 3) [12, 75, 76], Canada (*n* = 2) [77, 78], Finland (*n* = 1) [79], Greece (*n* = 1) [80], Israel (*n* = 1) [81] and Spain (*n* = 1) [82]. Most articles used Army personnel (*n* = 16, 40%) or Marines (*n* = 12, 30%). Articles included ‘regular’ personnel (*n* = 20, 50%), ‘cadets/recruits’ (in-training) (*n* = 15, 38%) and ‘reservists/conscripts’ (*n* = 5, 13%).

Force plates were primarily used to assess ‘performance’ (*n* = 31, 78%) or ‘injury profiling’ (*n* = 7, 18%). One article had a primary focus on informing ‘rehabilitation’ [12] and one article a combination of ‘injury profiling’ and ‘rehabilitation’ [48]. Across the 40 articles, 50 force plate tests were used. The most used tests included CMJ (*n* = 28, 70%), IMTP (*n* = 10, 25%) and squat jump (SJ) (*n* = 5, 13%). Other tests included DVJ (*n* = 2), single leg CMJ (*n* = 2), deadlift (*n* = 1) and leg press (*n* = 1). Most articles (*n* = 31, 78%) only used one force plate test to measure derivatives of maximal or rapid force production. The combination of two force plate tests were used in 8 articles [39, 53, 54, 56, 62, 71, 80, 81], with half (*n* = 4) using a combination of CMJ and IMTP [39, 53, 54, 56]. One article [69] used three tests that met the eligibility criteria.

### Force Plate Testing

Table 2 lists all force plate providers, reported sampling frequency and analysis software. Half of the articles (n = 20, 50%) did not report sampling frequency, and one-third (n = 13, 33%) did not specify how force–time data were processed.

### Isometric Force Plate Tests

Twelve articles (31%) used force plates to measure force production using an isometric task. The most used test was the IMTP (*n* = 10, 83%). Table 3 lists 23 methodological reporting considerations across 3 themes (preparation [*n* = 7], execution [*n* = 11] and analysis & reliability [*n* = 5]) when using force plates to measure force production during isometric tasks. When all 3 themes are combined, the mean count (indicating they have reported this methodological consideration) was 12.6 ± 4.7 (range 3 to 19) out of 23. Nevin et al. [75] reported the most complete methodology (count = 19, 83% complete), whereas Chasse et al. [77], Hamarsland et al. [62] and Poser et al. [57] reported the least (count between 3 and 8, or 13 to 35% complete) (see Table 3).

#### Preparation

Of the 7 methodological criteria reported for the ‘preparatory’ phase, the mean count across all 12 articles was 3.7 ± 1.5 (range 1 to 7) (Table 3). All articles reported the brand/manufacturer, and 8 articles (67%) reported sampling frequency of their force plate device. Only 2 articles (17%) reported zeroing the force plates at any point during test proceedings or reported a weighing phase prior to test execution. One article (8%) reported familiarisation sessions prior to testing. Ten articles (83%) reported usings a warm-up, and 9 articles (75%) reported practice attempts prior to maximal efforts/attempts.

#### Execution

Of the 11 methodological criteria reported for the ‘execution’ phase, the mean count taken across all 12 studies was 6.8 ± 2.8 (range 1 to 9) (Table 3). Most articles reported knee (*n* = 10, 83%) and hip angle (*n* = 8, 67%) but few reported handgrip position (*n* = 3, 25%) or footwear (n = 3, 25%). Lifting straps to support grip strength were reported in 9 articles (75%), and bar pre-tension was reported in 4 articles (33%). Verbal coaching cues/commands were described in 11 articles (92%). All articles reported the number of test repetitions,10 articles (83%) reported muscle contraction duration, and 9 articles (75%) specified recovery duration between attempts. Only 2 articles (17%) identified the test administrator.

#### Analysis and Reliability

Of the 5 methodological criteria reported for the ‘analysis’ phase, the mean count taken across all 12 studies was 2.2 ± 1.2 (range 0 to 4) (Table 3). Eleven articles (92%) reported data calculation methods, 6 articles (50%) defined the metrics and all reported statistical software used. However, only 7 articles (58%) named the software used to process force–time data (depicted with orange boxes in Table 3) and only one article [69] clearly defined filters being applied.

Reliability is rarely reported: Nevin et al. [75] reported excellent within- session reliability between repeated IMTP trials assessed via intraclass correlation coefficient (ICC = 0.96, 95% CI [0.94–0.98]). Hamarsland et al. [62] inferred reliability based on prior laboratory testing. Therefore, most articles (*n* = 10, 83%) did not report any reliability data. However, in a group of articles which share a similar cohort [39, 53, 54, 56], all discarded and repeated if deviation from acceptable force–time curve characteristics were observed, and an additional trial was performed if there was > 250 N difference in peak force between attempts.

#### Filters Applied and Cut-offs Used

Very little detail was provided regarding filters and cut-offs used during isometric testing. Doyle et al. [69] reported IMTP initiation was determined using a feature recognition algorithm that included a threshold requiring the force reading to be consistently greater than 5 N from the baseline force reading.

#### Risk of Bias

Among studies that utilised isometric force plate tasks, risk of bias was generally ‘low’ (*n* = 11, 92%) with one ‘moderate’ risk article [57].

### Methodological Reporting Practices of the Most Common Isometric Test: The Isometric Mid-Thigh Pull

The isometric midthigh pull (IMTP) was the most used force plate derived test (*n* = 10) for measuring isometric force production in military personnel. Of these 10 articles, 5 articles (50%) were conducted within an ‘occupational task performance’ context, 3 articles (40%) within an ‘injury profiling’ context, 1 article combined ‘occupational task performance and injury profiling’ and one article (10%) was conducted within a ‘rehabilitation’ context (see Table 4).

#### Test Execution, Verbal Cues and Test Administrator

Nine articles (90%) instructed participants to perform the IMTP using a knee flexion angle between 120–145°. One article [77] did not report knee flexion angle in their methodology. Eight articles (80%) reported a hip flexion angle between 140–150°. Two articles [69, 77] did not report the hip flexion angle. In the articles that did not report joint angles, neither described the IMTP posture as ‘the start of the second pull phase of the clean’. Instead, Doyle et al. [69] instructed participants to adopt a ‘semi-squat’ position. Most articles (*n* = 7, 70%) do not specify hand grip position on the bar, however, two articles reported a pronated grip position, and one article reported hand grip position being optional (not standardised). Eight articles used lifting straps to facilitate grip strength around the bar, thereby minimising the limitation of grip strength. One article [78] did not permit the use of lifting straps, and one article [77] did not report their use within their methodology. Six articles (60%) did not report any pre-tension being applied to the bar immediately prior to maximal effort, whereas 4 articles did instruct participants to apply some pre-tension (see Table 4).

Most instructed participants to “*pull* as hard and as fast as possible” (*n* = 7) or “*push/drive* their feet into the floor/ground as hard and as fast as possible” (*n* = 2). One article did not report any verbal instructions. Most articles (*n* = 8, 80%) did not mention who conducted the force plate assessments. Only Robitaille et al. [78] (fitness professional, medical technician, and registered physiotherapist) and Walters et al. [12] (biomechanist/research physiotherapist) reported who conducted the IMTP force plate assessments.

#### Footwear Condition

Only three articles reported footwear condition. McFadden et al. [39, 54] wore ‘athletic trainers’ and Petersen et al. [56] wore military boots.

#### Test Repetitions, Rest Duration, Duration of Test

All researchers used between 2 or 3 test repetitions. The duration of rest between attempts varied between 30 s and 2 min. Nine articles (90%) reported 5 s duration of maximal isometric muscle action/effort during each attempt.

#### Metrics Used

The number of force plate derived metrics reported from the IMTP test was not exhaustive (Supplementary File 4, Table S4). Half (*n* = 5) reported one metric only [39, 56, 76–78], 2 articles reported 2 metrics [54, 69], 2 articles reported 3 metrics [12, 75], and one article reported 4 metrics [53]. Peak force was the most frequently used metric (*n* = 8, 80%). However, within this metric four articles [69, 75, 77, 78] used the term ‘absolute’ peak force but did not define if this is gross force (i.e., including body mass) or net force (i.e., excluding body mass), one [12] used ‘net’ peak force and three articles [39, 54, 76] did not clearly define peak force (i.e., gross or net values), making comparisons between studies problematic. Relative peak force (N·kg^−1^) was also commonly reported (*n* = 6, 60%) although it is not always explicit if this was calculated from gross or net peak force. Two articles [53, 75] reported RFD but at different time intervals (0–100 and 0–250 m·s^−1^) with neither article clarifying whether they used peak or mean RFD, or a moving average window which has been shown to notably affect the magnitude and reliability of the values [83]. Limb symmetry (%) and time-to-peak force (secs) was recorded in one article only [53].

#### Summary of Force Plate Derived Strength Results

Comparing IMTP results between studies should be interpreted with caution as the 10 articles used 4 different force plate providers using very different protocols (preparation, execution and analysis) which have been shown to notably affect the subsequent values [83–87]. Some articles did not clarify whether they reported ‘gross’ or ‘net’ peak force values. However, average peak force values ranged between 1200 N (junior entry recruits) [76] to 3449 N (special forces candidates) [69]. Healthy personnel in regular/productive service reported mean peak force values between 1570 N (mix of men and women) [77] or 2417 N (men only) [75] to 3408 N (men only) [69]. In healthy recruits/cadets (aged over 18 years of age) this value is lower at 1340 N (mix of men and women) [76] or 1776 N (mostly men) [78] to 2626 N (men only) [39]. Sex differences in peak force were reported. Female values ranged between 1365 N [77] and 1747 N [54] and males ranged between 2417 N [74] and 3408 N [69]. As a comparison, the only ‘rehabilitation’ themed article reported net peak force values of 1281 N [12].

Relative peak force ranged between 17 N·kg^−1^ (in personnel undergoing rehabilitation) [12] to 38.4 N·kg^−1^ (in male US Marine Corps recruits) [56]. Healthy military personnel in ‘regular’ service reported mean relative peak force values of 28 N·kg^−1^ (men, from one article only) [75]. In healthy ‘recruits/cadets’ this value ranged between 24.5 N·kg^−1^ (women) to 38.4 N·kg^−1^ (men) both using US Marine Corps participants [56]. Sex differences were apparent. Relative peak force values for women ranged between 24.5 N·kg^−1^ to 30.6 N·kg^−1^ [56] and between 30.6 N·kg^−1^ to 38.4 N·kg^−1^ for men (all cited from the same article) [56].

### Dynamic Force Plate Tasks

Thirty-four articles (85% of the total) used force plates to measure dynamic force production capabilities. The most used test was the CMJ (*n* = 28, 82%) followed by SJ (*n* = 5, 15%), single limb CMJ (*n* = 2, 6%), DVJ (*n* = 2, 6%) and a stop jump (*n* = 1, 3%). A stop jump involved standing at a distance of 40% of body height away from the edge of the force plate, performing a double-limb broad jump to the force plates, landing with one foot on each plate, and immediately perform a maximal vertical jump [52].

Listed in Table 5 are 21 methodological reporting considerations across 3 themes (preparation [*n* = 8], execution [*n* = 7] and analysis [*n* = 6]) when using force plates to measure rapid dynamic force production. When all 3 themes are combined, the mean counts are 9.2 ± 3.4 (range 3 to 17) out of 21. Bird et al. [44, 45], and Kozinc et al. [72] reported most comprehensively (counts of 15–17 out of 21, or 71 to 81% complete). The least detail was provided by Conkright et al. [50], Vodičar et al. [74] and Yanovich et al. [81] (counts between 3 and 4 out of 21, or 14 to 19% complete).

#### Preparation

Of the 8 methodological criteria reported for the preparatory phase, the mean count across all 34 articles was 3.3 ± 1.5 (range 1 to 7) (Table 5). All articles reported force plate brand/manufacturer, but only 18 articles (53%) reported sampling frequency, 5 articles (15%) zeroed the force plates, 7 articles (21%) reported a weighing phase, and 2 articles (6%) described the surface/flooring conditions the force plates were place on. Familiarisation sessions were reported in 9 articles (26%), 27 articles (79%) reported a warm-up, and 11 articles (32%) reported practice attempts prior to maximal efforts being recorded.

#### Execution

Of the 7 methodological criteria reported for the ‘execution’ phase, the mean count taken across all 34 studies was 3.5 ± 1.5 (range 0 to 6) (Table 5). Twenty-two articles (65%) reported hand position/arm swing, and 13 articles (38%) reported joint angles/movement depth during the jumping task. Verbal cues/commands were given in 22 articles (65%). Most articles reported number of repetitions (n = 29, 85%) and rest periods between attempts (n = 23, 68%). Footwear condition was reported in 10 articles (29%). One article [46] reported footwear “being consistent” but did not specify if the force plate test was conducted wearing ‘athletic shoes’ or ‘military boots’). Only 3 articles (9%) reported who conducted force plate testing (see Table 5).

#### Analysis and Reliability

Of the 6 methodological criteria reported for the ‘analysis’ phase, the mean count taken across all 34 studies was 2.4 ± 1.4 (range 0 to 5) (Table 5). Most articles (*n* = 29, 85%) described data calculation and 17 articles (50%) defined metrics used. Filters were reported in 6 articles (18%). One article [48] reported filters applied to remove artifacts but gave no additional detail. Four articles (12%) clearly reported cut-offs used to identify contact. Most articles (*n* = 31, 91%) reported the software package used for statistical analysis, however, 8 articles (26%) did not specify the software used to process force–time data (depicted with orange and red boxes in Table 5). It should be noted that some software packages permit the user to alter the thresholds used to identify the onset of the movement, so it is imperative that this threshold is reported.

Only 2 articles (6%) [68, 72] reported cohort-specific reliability data. Debenedicts et al. [68] reported variable reliability depending on the metric used (ICC: time to peak landing = 0.84, peak landing force = 0.73, take off time = 0.62, rate of force production = 0.54). Kozinc et al. [72] reported relative reliability as high or excellent for all but one variable in both conditions (without equipment and with equipment) (ICC = 0.88–0.98). Four articles (12%) [46, 62, 64, 65] inferred reliability from previous laboratory work. Thus, reliability testing was not performed in 28 articles (82%).

#### Filters Applied and Cut-offs Used to Identify Contact

Filters and cut-offs were not commonly reported for dynamic tasks. Doyle et al. [69] reported that where necessary, a low-pass Butterworth filter with a trial specific cut offs were used for their CMJ and SJ testing. Johnson et al. [52] used a fourth order low-pass Butterworth filter, Kozinc et al. [72] used a 5 ms moving average filter before calculating their outcome variables of interest, Merrigan et al. [55] used a second order Butterworth low pass filter at 30 Hz and Zifchock et al. [60] applied a fourth order, 10 Hz Butterworth filter applied to all data used to ‘estimate’ peak power.

#### Risk of Bias

Most articles that utilised dynamic force plate tasks had a ‘low’ risk of bias (*n* = 29, 85%); 5 articles (15%) had a ‘moderate’ risk of bias.

### Methodological Reporting Practices of the Most Common Dynamic Test: Countermovement Jump

The CMJ was the most used dynamic force plate test to assess derivatives of muscle strength in military personnel. Twenty-eight of the 34 articles (82%) assessing ‘dynamic’ force production, or 70% of the total number of articles included within this review, used the CMJ. The test proved particularly popular when assessing large numbers of personnel; 16 articles recruited over 100 military personnel, 14 of these articles (87.5%) used the CMJ as a measure of force production (see Table 6).

Twenty-three articles (82%) used the CMJ within an ‘occupational task performance’ context and 6 articles (18%) within an ‘injury profiling’ context. No articles were found that used force plate technology to measure the CMJ to answer any ‘rehabilitation’ themed research questions.

### Test Execution, Verbal Cues and Test Administrator

Thirteen articles (46%) instructed participants to perform the CMJ with hands positioned on hips, 4 articles (14%) used an ‘arm swing’ and 10 articles (36%) did not report hand position/arm movement. Most articles (*n* = 17, 61%) did not report any instructions regarding joint angles or depth of movement. Six articles (21%) instructed ‘self-selected’ depth, 5 articles (18%) instructed participants to meet a specific countermovement depth prior to concentric effort (knee and hip joint flexion at ~ 90°). Six articles (21%) tested under ‘unloaded’ and ‘loaded’ conditions (typically wearing military boots, body armour and helmet) (see Table 6).

Nine articles (32%) instructed participants to “jump as high/maximally as you can”, 6 articles (21%) to “jump as high and as fast/explosively as possible” and 4 articles (14%) to jump immediately following a verbal “3–2-1” countdown. Nine articles (32%) did not report any verbal instructions. One article also provided instruction on landing, asking participants to “land with both feet striking the platform simultaneously”.

Only three articles (11%) identified test administrators. Bird et al. [48, 49] used a researcher and Solberg et al. [64] used a “qualified test personnel with Master’s degree in Sport Science”. Subsequently, 25 articles (89%) did not mention who conducted the force plate assessments.

#### Footwear Condition

Footwear was unreported in 17 articles (61%); reported footwear included military/combat ‘boots’ (n = 5, 18%), ‘athletic shoes’ (n = 3, 11%), ‘socks’ only (n = 1), ‘barefoot’ (n = 1) and combined ‘indoor running shoe’ and ‘boots’ for unloaded and loaded conditions (n = 1), respectively.

#### Test Repetitions and Rest Duration

Most articles used between 2 and 4 repetitions, with 16 articles (59%) reporting 3 attempts. Rest duration varied between 10 s and 2 min. Seven articles (26%) did not report rest duration between test attempts.

#### Metrics Used

Most articles reported one (*n* = 11, 39%) or two metrics (*n* = 6, 21%) only (see Supplementary File 4, Table S5). Three articles (11%) reported between 3 to 4 metrics, 6 articles (21%) reported 5 to 7 metrics, one reported 9 metrics, and one reported 14 metrics.

The most common metric reported was jump height recorded in cm (*n* = 19, 68%) followed by different expressions of power (peak power [*n* = 9, 32%], relative peak power [*n* = 6, 21%), average power [*n* = 3, 11%) and expressions of force (peak force [*n* = 3, 11%], relative peak force [*n* = 2, 7%], average force [*n* = 2, 7%] and impulse (*n* = 2, 7%). One article [53] reported metrics that define limb symmetry. Three articles (11%) reported metrics that define other phases of the CMJ, with modified reactive strength index (mRSI) being the most commonly used (*n* = 3). Five articles used Sparta Science force plates which had its own proprietary algorithms that reported non-standardised metrics such as ‘load’, ‘explode’ and ‘drive’ which are defined by Merrigan et al. [36] but not easily transferable to other studies across this review.

Of the 13 articles which reported different expressions of power during the CMJ, seven (54%) did not report which phase of the jump (e.g., eccentric/braking phase or concentric/propulsive phase) the power metric was derived from. Of the 6 articles which reported different expressions of force, four articles (67%) did not report the phase of the jump that the force metric was derived from.

#### Summary of Force Plate Derived Strength Results

Comparing differences in CMJ results between studies should be interpreted with caution as the 28 articles used 9 different force plate providers and used very different protocols (preparation, execution and analysis). However, average jump height values between articles ranged between 0.21 m (US female reservists) [50] to 0.44 m (special forces candidates) [69]. Healthy military personnel in regular/productive service reported mean jump height values between 0.22 m (women) [58] or 0.27 m (mix of men and women) [72] or 0.28 m (men only) [73] to 0.44 m (men only) [69]. In healthy recruits/cadets, this value was 0.21 m (women) [50] or 0.27 m (men) [50] to 0.42 m (men).

Sex differences were apparent with female jump height values ranging between 0.21 m [50] and 0.29 m [48, 65, 66] and men ranging between 0.27 m [50] and 0.44 m [69].

### Force Plate Derived Ratios

#### Dynamic Strength Index (DSI)

The dynamic strength index (DSI) is the ratio between the peak isometric force produced (for example, the IMTP) versus how much of that force is produced during a ballistic movement (for example, the CMJ), calculated as dynamic peak force divided by isometric peak force [88]. This enables the profiling of an individual’s “strength potential” and how much of this potential is being used during ballistic movements which are essential attributes when performing physically arduous military-specific tasks [11]. Only one article [56] reported DSI, stratifying male and female recruits (using k-means clustering) into two distinct clusters (high and low performers) with relevance to tactical and physical fitness. Men had lower DSI ratios in both groups (low performers, 0.77 ± 0.12; high performers, 0.62 ± 0.08) versus women (low performers, 0.93 ± 0.20; high performers, 0.73 ± 0.11). As an entire cohort they stratified low performers and high performers using DSI values of 0.82 ± 0.17 and 0.65 ± 0.10, respectively.

#### Eccentric Utilisation Ratio (EUR)

Eccentric utilisation ratio is calculated by dividing CMJ variables by the same SJ variables (e.g., jump height, take-off force, power, or flight time) to elicit an athletic profile, although this was originally meant to use jump height rather than other metrics. The CMJ includes an eccentric loading phase, which may allow for the utilisation of the stretch–shortening cycle [89]. In contrast, SJ starts with an individual in a static squat position, which may minimise the benefits of the stretch–shortening cycle to jump performance. Calculating EUR with these jumps may provide insight into an individual’s utilisation of eccentric muscle action to produce force, which is an indicator of training status and emphasis, that is, strength or power [90]. Doyle et al. [69] was the only study that reported EUR (peak CMJ force divided by peak SJ force). They did not define the phase of the CMJ or SJ jump where force was used within this calculation. A significant interaction of time and injury status was found using the EUR alongside significant correlations with preventable lower limb injury (*r* = 0.275, *p* = 0.014) and preventable knee injury (*r* = 0.227, *p* = 0.044) at baseline. Within this study, the uninjured group had lower EUR (0.94 ± 0.08, 1.01 ± 0.09, *p* = 0.025) compared to the injured.

## Discussion

Force plates are being used by military organisations to address diverse research questions. However, this review revealed substantial inconsistencies in the reporting of force plate methodological practices across ‘preparation’, ‘execution’ and ‘analysis’ between and within military organisations for both isometric and dynamic tasks. In circumstances where force plates added little insight [47, 49, 51, 71], authors relied on non-standardised metrics (e.g., load, explode and drive) from a single provider. In contrast, articles reporting standardised metrics generally contributed meaningfully to their research questions. This reinforces the importance of ensuring the force plate tests and metrics used have been validated against industry standards before being applied within real world settings [32].

### Methodological Standards

Describing the standardisation and methodological considerations for the IMTP, Comfort and colleagues [91] provided a list of recommendations that should permit more meaningful comparisons of individual performances between testing sessions, comparisons between individuals, and more effective comparisons between published studies. Their recommendations encouraged adjusting the bar height to achieve knee (125–145°) and hip (140–150°) angles which represent the start of the second pull phase of the clean. In addition, short familiarisation sessions, standardised warm-ups, sub-maximal practice attempts (e.g., 50%, 70% and 90% of perceived maximal effort), use of lifting straps, inclusion of a weighing phase (≥ 1 s quiet standing), and pre-tension to the bar are advised. Strong verbal encouragement should be applied with verbal cues such as ‘*drive/push* your feet into the floor as hard and as fast as possible’, not ‘*pull* the bar’. Additional trials should be repeated until peak force values differ by ≤ 250 N. It should be clear whether the force and/or impulse values reported are ‘absolute/gross’ or ‘net’ values. Thresholds for movement, any filtering/smoothing of data and sampling frequency should be specified [91] as this can notably affect temporal variables. Compared against these standards, most articles confirm hip and knee angle within acceptable ranges, reported a warmup, practice attempts and used lifting straps (≥ 80%). However, most articles did not report prior familiarisation session (92%), a weighing phase (83%) which is enforced by some force plate providers, or any pre-tension to the bar (67%). Whilst 90% reported strong verbal encouragement, the majority (80%) provided an incorrect coaching cue or no cueing at all. Only 2 articles (20%) instructed their participants to ‘*drive/push* their feet into the floor as hard and as fast as possible’, the majority instructed personnel to ‘*pull* the bar’. Only 40% of articles repeated trial attempts until peak force values of the trial were separated by ≤ 250 N, which would otherwise reduce the reliability and increase the variability between trials. Three articles [39, 54, 76] did not clearly define the peak force metric used (i.e., gross or net values), making comparisons between articles a problematic.

Additional shortcomings included not identifying who conducted the test (83%), zeroing the force plates (83%), mentioning hand grip positioning (e.g., supination, pronation or mixed grip—75%) and footwear condition (75%). Whilst Nevin et al. [75] controlled for most of these methodological considerations, the majority did not, or their verbal cueing was incorrect (negatively influencing the usability of some important metrics, such as RFD) [86, 92]. Consequently, most IMTP data collected and published using military personnel cannot be relied upon to accurately inform the end-user (military command, physical training staff, etc.) or the wider research community. Thus, the data reported within these selected articles should not be compared between or within military nations.

For dynamic tasks, the most common ‘preparation’ and ‘execution’ considerations not reported included, surface/flooring conditions (94%), tester identity (91%), force plate zeroing (85%), a weighing phase (79%), familiarisation (74%), footwear condition (71%) and practice attempts (68%). Filters and cut points for phase detection (for example, ground contact) were infrequently reported. When filters were applied, (e.g., 5 N from the baseline force reading) [69], this can be less than the residual noise from a portable force plate, challenging the interpretation of findings. All the above are believed to influence either the quality of data or values derived from force plates during maximum testing.

A lack of reliability testing is concerning. Such reporting practices are now mandatory across numerous biomechanics and strength and conditioning peer-review journals, thus suggesting most articles in this review might not be meeting minimum methodological reporting standards expected by the scientific community [93]. This is particularly pertinent to articles that reported pre and post (or multiple time points) measures. If the percentage error is near or comparable to the magnitude of change (pre to post) then findings are most likely not clinically meaningful. Unfortunately, most articles did not report reliability data which makes interpreting the findings (between baseline and follow-up measures) challenging. As a minimum within session reliability should be reported using intraclass correlation coefficients and the associated 95% confidence intervals (CI) interpreted based on the lower bound of the 95%CI [94], and percentage coefficient of variation [95]. However, between session reliability and measurement error would be ideal, if feasible.

The lack of standardisation and reporting of key methodological variables from both isometric and dynamic tasks and the lack of conducting reliability/error testing should be alarming to military researchers and applied practitioners, as these deficiencies hinder evidence-based policy and practice.

### Injury Profiling

Findings for injury profiling were mixed and appeared contingent on metric selection. Articles that used standardised force–time metrics found differences between MSKI and non-injured groups [48, 53, 69, 78], whereas those relying on non-standardised outputs (i.e., load, explode and drive) did not. [49, 51].

Using force plates in combination with motion capture analysis, Bird et al. [48] identified CMJ movement strategies clusters with differential MSKI risk in marine officer cadets (MOCs). The high-risk cluster had a relative risk of developing a MSKI 1.6 and 2.2 times higher than the moderate and low risk clusters, respectively. Absolute eccentric/braking RFD, eccentric/braking net impulse and concentric/propulsive net impulse, were higher in low-risk cluster groups compared to the high-risk cluster (p < 0.001). Lesser degrees of flexion and shorter CMJ phase durations (braking phase and propulsive phase) were observed in low-risk cluster compared to both moderate-risk and high-risk clusters. Male personnel were distributed equally across clusters while female MOCs were primarily distributed in the high-risk cluster. Lovalekar et al. [53] identified numerous modifiable risk factors for MSKIs during recruit training, including decreased relative peak concentric power, lower body mass index and cigarette use. There were several variables from the CMJ and IMTP that potentially predicted MSKI and lower limb MSKI. Notably, lower relative peak concentric power and shorter eccentric/braking deceleration duration were significant predictors of MSKIs (χ^2^ = 58.03, *p* < 0.001). Both Doyle et al. [69] and Robitaille et al. [78] found attributes of maximum strength to be protective against future MSKI occurrence. Specifically, Doyle et al. [69] found an association between baseline lower limb strength, as measured by force production and EUR, and MSKI. Robitaille et al. [78] developed a model including measures of absolute muscular strength (IMTP peak force) and cardiorespiratory fitness, together with self-reported previous history of MSKI to predict MSKI in two separate infantry cohorts.

### Rehabilitation

Although force plates had been advocated for return to duty decision support [31], only one article used force plate technology to assess maximum force production capability within a military rehabilitation setting [12]. Rehabilitation in military personnel aims to ensure that injured personnel can successfully meet the physical demands expected by their military unit. It is widely acknowledged that muscle strength (force production) forms the foundations of an individual’s ability to successfully complete most military tasks [1, 4–6, 26]. This is unsurprising as force relative to the mass being accelerated determines acceleration, based on Newton’s second law, and for complete deceleration to occur an impulse (impulse = force × time) the equivalent of an individual’s momentum (momentum = mass × velocity) is required. It is therefore surprising how few military rehabilitation researchers or applied practitioners have used force plates to measure derivatives of muscle strength to guide and inform rehabilitation practice. Instead, research groups have applied force plate technology to assess ‘stability’ during balance orientated tasks [96–99] or during gait assessments [97, 100–108]. These studies did not meet our inclusion criteria (inappropriate test and/or metric selection); however, they provide examples of how this technology can otherwise be applied to inform Defence rehabilitation practices.

### How Strong are Military Personnel?

Strength profiles varied widely, but poor and inconsistent methodological reporting precluded robust comparisons within and between militaries (or with athletic population]. This review highlights the need for a consensus on methodological reporting criteria to be agreed between international military research groups. The delivery of such a consensus would benefit from contributions of subject matter experts from within and outside of the military.

### Review Strengths and Limitations

Two researchers performed the entire process of search, selection, and extraction of data independently, thus increasing the accuracy of the data recorded and reduction of errors associated with this review process. In addition, we analysed the quality of each study, a task that is not necessary in a scoping review but performed to increase study rigor. We measured quality by analysing the risk of bias and those items that we considered necessary for a good methodological explanation of the tests/analyses.

Despite these strengths, it is acknowledged that the restriction by language (including only studies written in English) and publication type (only studies published in peer-reviewed journals) are limitations of our scoping review. However, we also believe these factors have not had a major impact on the conclusions drawn in our review. Whilst this review focussed on articles that use force plates to measure force production, another common application of this technology within the military ‘occupational task performance’ domain is to assess landing mechanics [109–117]. Our eligibility criteria excluded such articles (due to metric selection) and this may partly explain why most articles included in this review are ‘occupational task performance’ related. Researchers and applied practitioners should be mindful of this when selecting the most appropriate testing battery to meet the needs of their personnel and military unit. Subsequent research could include a more targeted systematic review (not scoping), focused on specific military populations or contexts, metrics, etc., to provide more precise insights into the use of force plates to inform military policy and practices.

### Knowledge Gaps and Future Recommendations

#### Standardising Reporting Procedures

An agreed checklist for preparation, execution and analysis is needed to standardise force plate assessments in military settings and improve data quality and confidence.

#### Force Plate Tests and Metrics

Despite their growing popularity and applied use with military personnel, when compared to examples from across elite sport [118–120]; Defence organisations do not appear to be maximally exploiting the number of tests and metrics that can be derived using force plate technology to better understand the force generating capacity of its people. Research into the use of force plate technology for the measurement of force production characteristics is still in its infancy for many military organisations (83% of the articles included in this review were published within the previous 5 years). Therefore, some military research groups and applied practitioners may not have a firm understanding of what insights a many of the metrics derived from force plate technology can offer their organisation. Therefore, they select the most widely used metrics from the available literature, which are predominantly outcome-based metrics (jump height or peak force) as opposed to strategy-based metrics. From a test selection perspective, numerous tests have not been investigated. From an isometric testing perspective, this includes multi-joint assessments, such as the isometric back squat and belt squat, and single-joint assessments such as the isometric hamstring assessments or isometric plantar flexion assessments. Whilst a few dynamic tests have been used (for example, the CMJ, SJ and DVJ), others have not, including the CMJ Rebound jump, 10/5 and 10/3 rebound, Scandinavian rebound, pogo jumps and Bosco jumps. Given some of the technical considerations associated with DVJ (including intra-individual variation between drop height and actual fall height) [121], some of these alternative rebound tests may prove more reliable at informing reactive force production qualities (such as reactive strength index). However, there is now a method of calculating fall height, rather than assuming fall height and box height are the same, built into one of the commercially available automated force plate systems, which may resolve this problem [121].

From a metrics perspective, there has been a greater emphasis in military research towards reporting ‘outcome’ (i.e., jump height) based metrics as opposed to ‘strategy’ metrics (e.g., countermovement depth, temporal variables). These ‘strategy’ metrics are more responsive to training [14] and fatigue [17, 18]. The consequences of this are that it is currently unclear which modifiable training variables (force and/or velocity) warrant greater attention in the physical preparation, injury mitigation or rehabilitation of military personnel.

This review found inconsistent use of terminology with many military research groups failing to report the phase of the jump where they calculated values such as force and power. This could be very problematic when trying to interpret study findings. The choice of metrics selected during the CMJ also warrants further scrutiny. Two of the most used metrics within the military literature include peak power and peak force. However, neither of these metrics determine jump height and could potentially be excluded in future research [8–10]. As an example, peak power and jump height are derived from peak velocity (based on impulse-momentum relationship). Therefore, the correlation between jump height and peak power is artificially inflated by the near-perfect correlation between jump height and the velocity at peak power [10]. Also, power can increase while jump height decreases and vice versa, based on changes in jump strategy. Pertinent to this review, jump strategy can be influenced by the verbal instruction/coaching cue used by the testing administrator. Within this review we found CMJ test instructions to be very inconsistent between articles. To inform jump strategy, components of impulse (phase duration and mean force during eccentric/braking and concentric/propulsive phases) may prove insightful to military organisations.

One study [56] aimed to determine whether k-means cluster analysis on baseline strength and power data derived from CMJ and IMTP partitions men and women who entered recruit training into distinct (high and low) performance clusters. This was the only study to report the use of the ratio-based metric DSI. A DSI value > 0.8 indicates that participants need to emphasise strength training and a value < 0.6 identifies that ballistic training should be emphasised. Using their entire cohort these authors stratified ‘low performers’ as 0.82 ± 0.17, and ‘high performers’ as 0.65 ± 0.10. However, without describing the component parts used to calculate DSI this is a too simplistic interpretation and the authors cannot make such classifications. For example, participant groups could have a DSI of 0.90, but if the participant group’s relative peak force is low then they will always be considered a ‘low performer’ in need of emphasising strength training, this is regardless of what the DSI value describes [122].

Often applied practitioners and research groups will use previously published peer-reviewed research as the rationale or justification for their choice of tests and/or selection of metrics used. Based on the findings from this scoping review, this practice must be scrutinised when using force plate technology to inform military populations. More transparency and detail surrounding force plate ‘preparation’, ‘execution’ and ‘analysis’ methodological reporting practices is urgently warranted. Frequently researchers used no more than two metrics from each test. For example, two or fewer metrics were used in 70% of IMTP and 60% of CMJ tests. Also, most articles (78%) only used one force plate test to determine the force generating capacity/profile of their population of interest. This highlighted an under exploitation of force plate technology to inform end user care and management, and inadequate detail being shared with senior leadership and command staff to inform current/future policy or practices on topics surrounding physical preparation, injury mitigation and rehabilitation.

#### Normative Data

From a physical preparation, injury profiling and rehabilitation perspective, understanding the optimal way to detect, treat and monitor those with or at risk of MSKI is essential if militaries are going to maximise the number of their personnel fit for operation [20]. For example, substantial clinical and research gaps exist within return to duty criteria for both the selection of metrics and an appropriate standard to be met along the return to duty care pathway [123]. To guide rehabilitation practice, physically prepare and develop injury mitigation strategies, depends on the knowledge of quality baseline normative datasets specific to the population of interest [7]. However, to provide such data, the testing methods and data analysis methods must be consistent [35, 36].

Benchmarking performance enables practitioners to better pinpoint an individual’s strengths and weaknesses that subsequently can be utilised in strength and conditioning programmes to identify components of fitness that require further attention. For example, the identification of below-average performances on the military fitness test or force plate test or metric. This involves understanding or quantifying the scale of typical scores for key assessment metrics (data distribution) and their measurement error (reliability bandwidth). This allows the practitioner to more accurately interpret individual assessment scores within a given session and monitor how they change over time. There is a lack of normative reference values of military personnel reported in the literature. Within this review, we identified one article [58] that collected normative reference values of US Marine Corps women performing the CMJ and presented their data using percentile distributions (i.e., 5th, 10th, 25th 50th, 75th, 90th and 95th percentiles). Whilst informative, alternative methods of presenting normative data (to indicate high and low performers within a group) have been advised. This includes calculating and comparing standard scores (i.e., inter-individual: Z-scores, Sten scores, and percentile rankings) [14]. Useful examples of how to present normative data from elite sport literature include Berberet et al. [118], Soriano et al. [119] and McMahon et al. [120]. The collection of normative datasets could offer military organisations enormous insights into screening (benchmarking) occupational task performance. Examples include the start and end of recruit training, injury profiling and guiding late-stage rehabilitation practice of military personnel in productive service [7]. Benchmarking could also be used as a pre-screening tool to identify individuals at increased risk of injury or failing to complete arduous training courses (such as special forces selection). For example, should a training establishment screen/profile 2,000 personnel on day one of an arduous course (over multiple course intakes) they will be able to determine the minimum thresholds of maximal and rapid force production characteristics required to pass specific milestones along the course. For example, they might have determined that 35 to 40 N.kg of relative peak force in the IMTP is the minimum standard required to pass the most physically demanding section of the course. If personnel arrive at a pre-selection review several months prior and are only able to produce 25 N.kg^−1^ then the training team will have an increased degree of confidence that this individual is physically unprepared to attend the next course and should be encouraged to return to their unit to train on maximal strength deficits.

#### Participant Sex

Despite considerable research effort investigating the introduction of women in ground close combat roles in the past decade, only one article recruited exclusively women. Whilst the recruitment of 27% women as a demographic might be considered ‘under-reporting’, in most military nations, women make up less than 20% of military personnel. Nevertheless, there is wide potential for integrating force plate technology to better understand the force generating capacity of women in different job roles from benchmarking occupational task performance, exercise feedback, fatigue monitoring, injury profiling, guiding rehabilitation and whether alternative physical development strategies are warranted compared to their male counterparts. However, based on the differences in stature between men and women it is important to ensure that metrics are scaled to body mass, where appropriate, to ensure that differences in performance are not simply a product of differences in relative strength [124–126].

#### Age

There is a growing emphasis on the importance of ‘retention strategies’ across military organisations. One theme of this relates to maintaining the physical health and performance of long-serving personnel, so their respective organisations can benefit from their experience and leadership attributes [1]. Maintaining the physical health and performance of this demographic is particularly valuable for Operational Leadership and Command. In terms of maximally exploiting the use of force plate technology to military organisations, a substantial knowledge gap exists in the physical health and performance of men and women with a mean age > 35 years. One example associated with the importance of collecting data in this demographic is the monitoring of joint health throughout Service careers, particularly following a traumatic MSKI. A need to develop secondary prevention strategies to mitigate or reduce the risk of post-traumatic osteoarthritis following MSKI and potential medium to long term medical downgrading is recognised [123]. Force plate derived physical assessments may offer greater insights into force generating capacity of military personnel helping to prevent, detect, and treat future MSKI related health concerns [127].

## Conclusion

The use of force plate technology to measure force production capabilities among military personnel is growing fast. However, this review has highlighted large inconsistencies in the methodological reporting standards of force plate assessments used to measure lower-limb force production in military settings and across nations. If military organisations are to effectively use force plate technology to inform or guide physical preparation strategies (occupational task performance), injury mitigation strategies (injury profiling) or rehabilitation practices, greater awareness of variables that influence the quality/accuracy of data outputs is warranted. Due to most articles not controlling for important variables associated with force plate ‘preparation’, ‘execution’ or ‘analysis’, confidence in the accuracy of data published to date is challenging. Therefore, meaningful comparisons of individual performances between testing sessions, comparisons between individuals/groups, and more effective comparisons between published studies are not advised based on the existing literature published within military settings.

## Supplementary Information

Supplementary Material 1.

Supplementary Material 2.

Supplementary Material 3.

Supplementary Material 4.

## Data Availability

All data generated or analysed during this study are included in this published article [and its supplementary information files].

## Abbreviations

CMJ: Countermovement jump
DMS: Defence Medical Services
DSI: Dynamic strength index
DVJ: Drop vertical jump
EUR: Eccentric utilisation ratio
IMTP: Isometric midthigh pull
MSKI: Musculoskeletal injury
RFD: Rate of force development
SJ: Squat jump
TTT: Time to take-off
