# Supplementary material for: The Use of Force Plate Technology to Measure Force Production Characteristics in Military Personnel: A Scoping Review of Methodological Reporting Practices
Source: Sports Med Open. 2025 Nov 21;11:140. doi: 10.1186/s40798-025-00942-6 (PMC12638576; doi:10.1186/s40798-025-00942-6)
Supplement: Supplementary file 2 — Supplementary Material 2. [file 40798_2025_942_MOESM2_ESM.docx]

| **Supplementary file 2.**  **Table S2:** Rational for inclusion/exclusion criteria for literature search. | | | |
| --- | --- | --- | --- |
|  | **Inclusion criteria** | **Exclusion criteria** | **Rationale for these criteria** |
| Population | Military personnel (recruits/cadets, regular productive service, reservists/conscripts). | Veterans. | The force plate methodologies used to assess the fitness and physical performance of military personnel at high risk of musculoskeletal injuries will be the primary outcome of interest. Thus, only those athletes training and competing regularly will be studied. |
| Concept | Force plate methodologies, tests, and metrics applied to assess physical performance, injury profiling or rehabilitation process. | Tests using equipment other than force plates. Metrics collected via other technology (e.g., 3-D motion analysis systems). | The aim of this scoping review is to summarise the force plate methodologies which are being implemented across different military settings. Therefore, only tests and metrics carried out on or extracted from force plates will be included. |
| Context | Primary, secondary and/or tertiary injury prevention. In studies focused on primary prevention, a measure of the injury occurrence should be reported. | Other study and assessment purposes. Any study that does not report injuries. | Based on the aim of this scoping review, only studies focused on injury profiling and rehabilitation will be included. Other contexts where force plates are used, such as those with physical performance purposes, will be excluded. |
| Publication type | Peer-reviewed original research articles only. | Non-peer-reviewed articles (i.e., pre-print documents), newspapers, opinion pieces, systematic reviews and meta-analysis, editorials, commentaries and letters to the editor, conference proceedings/abstracts, and book chapters. | For reasons of practicality and to avoid duplication of data, it is deemed acceptable to include only studies published in peer-reviewed journals. |
| Language | English. | Non- English. | According to the researchers’ proficiency, it is deemed acceptable to include only studies published in English. |
| Study design | Quantitative study designs, including randomized controlled trials, non-randomized controlled trials, quasi-experimental, before and after studies, prospective and retrospective cohort studies, case-control studies, and cross-sectional studies. | Qualitative studies and literature reviews. | To provide a comprehensive overview of the existing evidence, a broad range of quantitative study designs will be included. |
| Publication date | The date for the last update of the systematic search will be as close as possible to the date of completion of the first draft of the study. | N/A | All articles will be included regardless of the time period. |

**Table S2:** Medline Search terms/criteria

| **Search ID#** | **Search Terms** |
| --- | --- |
| S33 | S25 OR S28 OR S31 |
| S32 | S25 OR S28 OR S31 |
| S31 | S3 AND S30 |
| S30 | TI ( “Force plate” OR “force platform” ) OR AB ( “Force plate” OR “force platform” ) |
| S29 | S3 AND S24 |
| S28 | S3 AND S13 AND S24 |
| S27 | S3 AND S11 AND S19 |
| S26 | S3 AND S13 AND S19 |
| S25 | S3 AND S13 AND S19 AND S24 |
| S24 | S20 OR S21 OR S22 OR S23 |
| S23 | AB “Force plate” OR “force platform” OR “kinetic analysis” OR biomechanics OR “neuromuscular performance” OR isometric OR ballistic OR jump OR hop OR squat OR “mid-thigh pull” OR “mid thigh pull” OR “peak force” OR “force development” OR “reactive strength index” OR “limb symmetry” OR “muscle strength” |
| S22 | TI “Force plate” OR “force platform” OR “kinetic analysis” OR biomechanics OR “neuromuscular performance” OR isometric OR ballistic OR jump OR hop OR squat OR “mid-thigh pull” OR “mid thigh pull” OR “peak force” OR “force development” OR “reactive strength index” OR “limb symmetry” OR “muscle strength” |
| S21 | MM "Muscle Strength" |
| S20 | MM "Biomechanical Phenomena" |
| S19 | S14 OR S15 OR S16 OR S17 OR S18 |
| S18 | AB Pre-intervention OR “pre intervention” OR preintervention OR post-intervention OR “post intervention” OR postintervention OR “functional status” OR “physical function” OR “exercise test*” OR “physical standard*” OR screening OR assessment OR “performance profiling” OR benchmarking OR deployment |
| S17 | TI Pre-intervention OR “pre intervention” OR preintervention OR post-intervention OR “post intervention” OR postintervention OR “functional status” OR “physical function” OR “exercise test*” OR “physical standard*” OR screening OR assessment OR “performance profiling” OR benchmarking OR deployment |
| S16 | MM "Benchmarking" |
| S15 | MM "Exercise Test" |
| S14 | MM "Functional Status" |
| S13 | S4 OR S5 OR S6 OR S7 OR S8 OR S9 OR S10 OR S11 OR S12 |
| S12 | AB Rehabilitation OR physiotherapy OR “physical therapy” OR “physical activity” OR Pre-surgery OR Presurgery OR “pre surgery” OR pre-operative OR Preoperative OR ”pre operative” OR post-surgery OR postsurgery OR “post surgery” OR Post-operative OR Postoperative OR “post operative” OR “return to running” OR “back into running” OR “return to work” OR training OR exercise OR strength OR plyometrics OR resistance OR endurance OR fitness OR “injury prevention” OR “risk factor” |
| S11 | TI Rehabilitation OR physiotherapy OR “physical therapy” OR “physical activity” OR Pre-surgery OR Presurgery OR “pre surgery” OR pre-operative OR Preoperative OR ”pre operative” OR post-surgery OR postsurgery OR “post surgery” OR Post-operative OR Postoperative OR “post operative” OR “return to running” OR “back into running” OR “return to work” OR training OR exercise OR strength OR plyometrics OR resistance OR endurance OR fitness OR “injury prevention” OR “risk factor” |
| S10 | (MM "Risk Factors") |
| S9 | (MM "Physical Fitness") |
| S8 | (MM "Plyometric Exercise") |
| S7 | (MM "Exercise") |
| S6 | (MM "Return to Work") |
| S5 | (MM "Physical Therapy Modalities") |
| S4 | (MM "Rehabilitation") |
| S3 | S1 OR S2 |
| S2 | AB Military OR “Armed Forces” OR Navy OR Naval OR Army OR “Air Force” or Marines OR “Special Forces” |
| S1 | TI Military OR “Armed Forces” OR Navy OR Naval OR Army OR “Air Force” or Marines OR “Special Forces” |

**CINAHL search strategy**

| **Search ID#** | **Search Terms** | **Search Options** |
| --- | --- | --- |
| S34 | S28 OR S30 OR S32 | Limiters: English Language |
| S33 | S28 OR S30 OR S32 |  |
| S32 | S3 AND S25 |  |
| S31 | S3 AND S27 |  |
| S30 | S3 AND S13 AND S27 |  |
| S29 | S3 AND S13 AND S19 |  |
| S28 | S3 AND S13 AND S19 AND S27 |  |
| S27 | S20 OR S21 OR S22 OR S23 OR S24 OR S25 OR S26 |  |
| S26 | AB (“Force plate” OR “force platform” OR “kinetic analysis” OR biomechanics OR “neuromuscular performance” OR isometric OR ballistic OR jump OR hop OR squat OR “mid-thigh pull” OR “mid thigh pull” OR “peak force” OR “force development” OR “reactive strength index” OR “limb symmetry” OR “muscle strength”) |  |
| S25 | TI (“Force plate” OR “force platform” OR “kinetic analysis” OR biomechanics OR “neuromuscular performance” OR isometric OR ballistic OR jump OR hop OR squat OR “mid-thigh pull” OR “mid thigh pull” OR “peak force” OR “force development” OR “reactive strength index” OR “limb symmetry” OR “muscle strength”) |  |
| S24 | (MM "Muscle Strength") |  |
| S23 | (MM "Squatting") |  |
| S22 | (MM "Hopping") |  |
| S21 | (MM "Jumping") |  |
| S20 | (MM "Biomechanics") |  |
| S19 | S14 OR S15 OR S16 OR S17 OR S18 |  |
| S18 | AB (Pre-intervention OR “pre intervention” OR preintervention OR post-intervention OR “post intervention” OR postintervention OR “functional status” OR “physical function” OR “exercise test*” OR “physical standard*” OR screening OR assessment OR “performance profiling” OR benchmarking OR deployment) |  |
| S17 | TI (Pre-intervention OR “pre intervention” OR preintervention OR post-intervention OR “post intervention” OR postintervention OR “functional status” OR “physical function” OR “exercise test*” OR “physical standard*” OR screening OR assessment OR “performance profiling” OR benchmarking OR deployment) |  |
| S16 | (MM "Benchmarking") |  |
| S15 | (MM "Exercise Test") |  |
| S14 | (MM "Functional Status") |  |
| S13 | S4 OR S5 OR S6 OR S7 OR S8 OR S9 OR S10 OR S11 OR S12 |  |
| S12 | AB (Rehabilitation OR physiotherapy OR “physical therapy” OR “physical activity” OR Pre-surgery OR Presurgery OR “pre surgery” OR pre-operative OR Preoperative OR ”pre operative” OR post-surgery OR postsurgery OR “post surgery” OR Post-operative OR Postoperative OR “post operative” OR “return to running” OR “back into running” OR “return to work” OR training OR exercise OR strength OR plyometrics OR resistance OR endurance OR fitness OR “injury prevention” OR “risk factor*”) |  |
| S11 | TI (Rehabilitation OR physiotherapy OR “physical therapy” OR “physical activity” OR Pre-surgery OR Presurgery OR “pre surgery” OR pre-operative OR Preoperative OR ”pre operative” OR post-surgery OR postsurgery OR “post surgery” OR Post-operative OR Postoperative OR “post operative” OR “return to running” OR “back into running” OR “return to work” OR training OR exercise OR strength OR plyometrics OR resistance OR endurance OR fitness OR “injury prevention” OR “risk factor*”) |  |
| S10 | (MM "Risk Factors") |  |
| S9 | (MM "Physical Fitness") |  |
| S8 | (MM "Plyometrics") |  |
| S7 | (MM "Exercise") |  |
| S6 | (MM "Job Re-Entry") |  |
| S5 | (MM "Physical Therapy") |  |
| S4 | (MM "Rehabilitation") |  |
| S3 | S1 OR S2 |  |
| S2 | AB (Military OR “Armed Forces” OR Navy OR Naval OR Army OR “Air Force” or Marines OR “Special Forces”) |  |
| S1 | TI (Military OR “Armed Forces” OR Navy OR Naval OR Army OR “Air Force” or Marines OR “Special Forces”) |  |

**EMBASE search strategy**

1     (Military or "Armed Forces" or Navy or Naval or Army or "Air Force" or Marines or "Special Forces").ti. (33880)
2     (Military or "Armed Forces" or Navy or Naval or Army or "Air Force" or Marines or "Special Forces").ab. (80449)
3     1 or 2 (92343)
4     *rehabilitation/ (38266)
5     *physiotherapy/ (30534)
6     *return to work/ (2806)
7     *exercise/ (135874)
8     *strength/ (1470)
9     *plyometrics/ (717)
10     *endurance/ (7935)
11     *fitness/ (17341)
12     *risk factor/ (136572)
13     (Rehabilitation or physiotherapy or "physical therapy" or "physical activity" or Pre-surgery or Presurgery or "pre surgery" or pre-operative or Preoperative or "pre operative" or post-surgery or postsurgery or "post surgery" or Post-operative or Postoperative or "post operative" or "return to running" or "back into running" or "return to work" or training or exercise or strength or plyometrics or resistance or endurance or fitness or "injury prevention" or "risk factor").ti. (1127276)
14     (Rehabilitation or physiotherapy or "physical therapy" or "physical activity" or Pre-surgery or Presurgery or "pre surgery" or pre-operative or Preoperative or "pre operative" or post-surgery or postsurgery or "post surgery" or Post-operative or Postoperative or "post operative" or "return to running" or "back into running" or "return to work" or training or exercise or strength or plyometrics or resistance or endurance or fitness or "injury prevention" or "risk factor").ab. (4405722)
15     4 or 5 or 6 or 7 or 8 or 9 or 10 or 11 or 12 or 13 or 14 (4779638)
16     *functional status/ (11139)
17     *physical performance/ (8707)
18     *exercise test/ (15964)
19     *benchmarking/ (4424)
20     (Pre-intervention or "pre intervention" or preintervention or post-intervention or "post intervention" or postintervention or "functional status" or "physical function" or "exercise test*" or "physical standard*" or screening or assessment or "performance profiling" or benchmarking or deployment).ti. (778261)
21     (Pre-intervention or "pre intervention" or preintervention or post-intervention or "post intervention" or postintervention or "functional status" or "physical function" or "exercise test*" or "physical standard*" or screening or assessment or "performance profiling" or benchmarking or deployment).ab. (2613787)
22     16 or 17 or 18 or 19 or 20 or 21 (3005903)
23     *biomechanics/ (34337)
24     *isometrics/ (514)
25     *muscle strength/ (18791)
26     ("Force plate" or "force platform" or "kinetic analysis" or biomechanics or "neuromuscular performance" or isometric or ballistic or jump or hop or squat or "mid-thigh pull" or "mid thigh pull" or "peak force" or "force development" or "reactive strength index" or "limb symmetry" or "muscle strength").ti. (36755)
27     ("Force plate" or "force platform" or "kinetic analysis" or biomechanics or "neuromuscular performance" or isometric or ballistic or jump or hop or squat or "mid-thigh pull" or "mid thigh pull" or "peak force" or "force development" or "reactive strength index" or "limb symmetry" or "muscle strength").ab. (168265)
28     23 or 24 or 25 or 26 or 27 (212007)
29     3 and 15 and 22 and 28 (186)
30     3 and 15 and 22 (4556)
31     3 and 13 and 22 (1160)
32     3 and 13 and 28 (371)
33     3 and 28 (1360)
34     3 and 13 and 28 (371)
35     29 or 34 (470)
36     limit 35 to english language (452)
