# Supplementary material for: The Use of Force Plate Technology to Measure Force Production Characteristics in Military Personnel: A Scoping Review of Methodological Reporting Practices
Source: Sports Med Open. 2025 Nov 21;11:140. doi: 10.1186/s40798-025-00942-6 (PMC12638576; doi:10.1186/s40798-025-00942-6)
Supplement: Supplementary file 3 — Supplementary Material 3. [file 40798_2025_942_MOESM3_ESM.docx]

**Supplementary Table 3: Summary of aims and conclusions from each included article**

| **First Author** | **Study Aim** | **Force Plate Related Conclusions** |
| --- | --- | --- |
| Angelviet et al [61] | Investigating performance variables that can predict performance during a casualty evacuation drill. | Maximal power in the countermovement jump (CMJ) was correlated with performance in the evacuation test. |
| Barrett, et al. [47] | To profile physical performance prior to and throughout a selection course, with the aim of predicting success/failure from course. | A pre-training force plate assessment performed did not predict graduation rates. For Marines who successfully completed the course, Sparta scores serial measures (i.e., load, explode and drive) obtained throughout training were highly variable for each individual and no firm conclusions could be drawn related to load imposed or the fitness attained during training. |
| Bird et al. [48] | Evaluate movement strategies of the countermovement jump (CMJ) in Marine officer candidates via marker less motion capture (mMoCap) and force plate technology by clustering variables to create distinct movement strategies associated with MSKI sustained during Officer Candidates School. | CMJ movement strategies are associated with MSKI risk in military populations. By utilizing unsupervised cluster analysis techniques, three distinct CMJ movement strategies were identified that differed in the proportion of personnel with a MSKI. The high-risk cluster had a relative risk of developing a MSKI 1.6 and 2.2 times higher than the moderate and low risk clusters, respectively. |
| Bird et al. [49] | Evaluate the SPARTA™ and DARI™ composite scores in male and female Marine Officer Candidates in association with lower extremity and torso MSKI’s during 10-weeks of Marine Corps Officer Candidates training. | Composite SPARTA™ (MSKI Health score) and DARI™ (Readiness score and Performance score) are predictive of MSKI, but with limited clinical relevance due to the poor discrimination area under the curve (AUC) performance (AUC = 0.55–0.57). Composite scores and component population specific models were poor predictors of MSKI in candidates. Single composite scores and a trained model of the normalized component scores have limited utility to predict MSKI over 10-weeks of Officer Candidates School in Marines. |
| Burley et al. [67] | To determine the effect of a novel low volume high intensity concurrent training regimen and warm-up on physiological performance and musculoskeletal injury in Australian recruits. Force plates were used to assess squat jump performance, an outcome measure used to assess the efficacy of the training intervention. | physical performance benefits can be attained within a military training environment when participants are engaged in a progressive, and individualised concurrent training regimen, that consists of a specific functional warm-up, low volume high intensity interval running and functional compound specific strength exercise. and was associated with reduced musculoskeletal injury. |
| Chassé et al. [77] | Determine what modifiable and non-modifiable physical and physiological characteristics (using IMTP) contribute to the performance of an urban operation casualty evacuation and its predictive test (FORCE combat) in female soldiers. | Body composition and maximal strength are important contributors to the performance on an urban operation casualty evacuation in both males and females. (lean body mass to dead mass ratio (LBM:DM) and IMTP peak force predicted around 74% of casualty evacuation performance - IMTP was a significant predictor. |
| Conkright et al. [50] | Impact of exposure to a 5-day simulated military operational stress on physical performance, using the CMJ. | Men achieved higher values than women across the protocol in most tests, including vertical jump height. Men and women military personnel experienced similar changes in occupationally relevant tactical mobility performance. Acute occupational stress resulted in no differences in physical performance responses. |
| Debenedictis et al. [68] | Determine whether exposure to whole body vibration (WBV) from spending excessive amounts of time in vehicles reduces occupation specific physical performance measures, using drop vertical jump. | Exposure to WBV experienced during motorised land transit has a negative influence on aspects of lower body explosive strength (drop vertical jump performance. Practical implications: Exposure to two hours of simulated military land transit negatively impacted lower limb muscular power. |
| Doyle et al. [69] | First, identify if performance measures could elucidate relationships between strength and power qualities (IMTP, CMJ, squat jump, eccentric utilization ratio [EUR]) and future injury occurrence. Second, monitor strength and power qualities changes during a selection course, and if changes differed between uninjured and injured candidates. | An association was found between baseline lower limb strength, as measured by force production and eccentric utilisation ratio, and injury. This indicates that high levels of maximal strength may be protective against preventative lower limb injuries, particularly at the knee. |
| Groeller et al. [70] | Pre-post basic military training and initial employment training measures of physical performance in Australian Army soldiers. | Vertical jump height did not change throughout basic military training or initial employment training. |
| Hamarsland et al. [62] | First, to investigate the effect of an extremely demanding 1-week military training course on physical performance, body composition and blood biomarkers among apprentices to the Norwegian Naval Special Operations Command. Second to examine the recovery of these variables up to 2 weeks after the course. | CMJ performance and maximal isometric leg press performance dropped by approximately 30% and 20% respectively over the 1-week course, and CMJ did not recover within 2 weeks of rest. |
| Hando et al. [51] | Determine the association between scores from Sparta Science force plate scans and the risk of MSKI in US Air Force Special Warfare trainees during an 8-week training course. Furthermore, the test-retest reliability of the Sparta scores was evaluated in a sub-study. | Overall, 308 (36.4%) trainees had an MSKI during the surveillance period. However, no significant associations were found between the proprietary force plate vertical jump scan output scores and the likelihood of experiencing either an MSKI or a lower extremity MSKI. All force plate output scores demonstrated excellent test-retest reliability (ICC 0.90). |
| Johnson et al. [52] | Determine if military personnel with chronic low back pain have reduced performance, and if they use a different strategy to complete a functional performance task as compared to healthy military personnel. | This study demonstrates that active-duty United States Marine Corps Forces Special Operations Command personnel with a history of low back pain reach similar levels of jump height during a counter movement jump, as compared to those without a history of low back pain. However, the asymmetries displayed by the low back pain group suggest an alternate strategy to reaching similar jump heights as compared to healthy individuals. |
| Karatrantou et al. [80] | compared the efficacy of two whole-body vibration (WBV) protocols with equal training volume and different frequency of training sessions/week on body composition and physical fitness (neuromuscular performance, lower body strength and power, and aerobic/anaerobic fitness). | Short-term vertical simultaneous WBV training performed at high frequency is an effective and time-efficient mode of exercise for reducing body fat and for improving flexibility, jumping performance and maximal strength of lower limbs in young, physically active individuals. |
| Kozinc et al. [72] | First, assess intrasession reliability of biomechanical outcomes obtained from CMJ with and without military equipment. Second, assess the sensitivity of the CMJ outcomes to the effect of a 12-week resistance exercise intervention in members of Slovenian Armed Forces. | This study confirmed the high to excellent reliability of CMJ with and without military equipment. The 12-week full body resistance training elicited modest improvements in CMJ height, with slightly higher effects seen in CMJ with equipment. Performing vertical jumping assessments with military personnel wearing their equipment is reliable and could perhaps represent a more ecologically valid alternative to bodyweight CMJ. |
| Lovalekar et al. [53] | The purpose of this study was to examine the demographic, psychological, and physiological risk factors for MSKIs and attrition during US Marine Corps recruit training.  Can metric derived from force plate tests be used as risk factors for future MSKI and attrition during recruit training. | This study identified numerous modifiable risk factors for MSKIs and attrition during recruit training, including decreased muscular power. Important finding - there was a number of variables from the CMJ and IMTP that predicted MSKI and lower limb MSKI. |
| McFadden et al. [54] | Monitor US Marines throughout recruit training and to assess and compare workload, sleep, stress, and performance responses between sexes. | Both men and women experienced similar declines in power performance in the CMJ between week 2 and week 11. There were minimal changes in strength outputs from the IMTP. |
| McFadden et al. [39] | Evaluate training demands and characteristics that were associated with performance outcomes during the 13-week US Marine recruit training program. | Weak to moderate correlations were found between workload variables and changes in performance metrics. Increases in body mass throughout training were positively associated with strength and power changes. Changes in peak power were related to improvements in combat fitness test scores; however, higher workloads (i.e., energy expenditure) were negatively associated with peak power. |
| Merrigan et al. [46] | Evaluate the change in unloaded and loaded CMJ force-time characteristics across a competitive high-intensity training event in active-duty Marines. | 20 kg loaded CMJs are stable neuromuscular measures suitable for tracking chronic training adaptations. Monitoring unloaded and 10 kg loaded CMJ performances, along with movement strategies (i.e., countermovement rates and depth), may help identify moments of accumulated fatigue to inform training and recovery adjustments and improve the sustainability of personnel. |
| Merrigan et al. [55] | Examine effects of sex, drop height, and external load on drop jump kinetics and kinematics in ROTC cadets and to determine whether findings were associated with knee extensor and flexor strength. | The greater forces that occurred during 60 cm unloaded compared with 30 cm unloaded were not explained by any strength variable. The findings indicate that 15 kg loads had little influence on drop jump kinetics. These findings would suggest that sex is not a risk factor when performing drop jump tasks with external loads or from greater heights, but instead absolute strength levels may indicate risk. Thus, females in the current study seem to adapt strategies, such as landing with faster knee velocities, because of physiological differences, including reduced muscle stiffness and lower mechanical efficiency during eccentric actions compared with males. |
| Nevin et al. [75] | First, assess the relationship between isometric force-time characteristics and 2 km loaded (25 kg) march performance. Second, examine the relationship between isometric force-time characteristics and standing long jump performance. | Absolute peak force, relative peak force, and rate of force development (0–250 ms) demonstrated a small correlation with 2 km loaded march performance. Conversely, relative isometric peak force demonstrated a large correlation with Standing long jump distance. Collectively, these findings suggest that although the IMTP may be a reliable and valid measure of strength, the relationship between isometric force-time characteristics and load carriage performance seems to be limited. |
| Øfsteng et al. [63] | Investigate the effect of 10-day military field exercise with severe energy deficit on changes in body composition, endocrine responses, and physical performance in soldiers. | 10 days of military exercise with HIGH intake of protein and low intake of carbohydrate led to similar decreases in physical performance as LOW intake of protein and low intake of carbohydrate. Physical performance was measured as countermovement jump height, maximal strength, and cycling sprint power. After 7 days of recovery, most variables had returned to close to pre-exercise levels, except for CMJ, which remained at reduced levels, suggesting impaired stretch-reflex functionality. |
| Orantes-Gonzalez et al. [82] | Determine which of the following important variables influences performance the most during an obstacle test in which soldiers do and do not carry combat equipment: aerobic capacity, body composition or leg strength. | The jump height was not a significant performance factor. |
| Peterson et al. [56] | First, determine whether k-means cluster analysis on baseline strength and power data derived from CMJ and IMTP partitions men and women entering recruit training into distinct performance clusters. Second, assess the between-cluster incidence of injury. | K-means cluster analysis for measures of strength and power was effective in partitioning clusters of recruits on characteristics of strength and power. Subsequent analysis determined that the clusters were also distinct in terms of performance in the combat fitness test and physical fitness test. |
| Pihlainen et al. [79] | Evaluate the associations of physical fitness and body composition characteristics with simulated high-intensity military task performance. | Muscular power of the lower extremities seems to be a good predictor of high-intensity military performance, The 2 statistically strongest variables, which were inversely associated with the military simulation test performance, were dead mass ratio and CMJ performed in combat load. Furthermore, all variables measuring muscle power of the lower extremities were good predictors of performance. In this study, Standing Long Jump (SLJ) produced nearly similar correlations as CMJ did with the Military task time. In addition, the correlation between CMJ and SLJ was high. |
| Poser et al. [57] | Examine if isometric peak force and rate of force development (RFD) were related to the ability to successfully perform a simulated casualty evacuation task in both unweighted and weighted conditions. | On average, individuals who achieved an isometric deadlift peak force of 1420 N (equivalent to 144 kg) or above were able complete a simulated casualty evacuation task with additional duty gear load (18 kg total). This provides preliminary data for the development of a strength cut point that can be refined with further high-fidelity task simulations to determine if military personnel are strong enough to complete this basic but influential task. |
| Potter et al. [58] | Characterise the body composition and physical performance of elite women war fighters. | There is a low correlation between relative body fat and performance measures of these Marine women. Body composition is a poor predictor of physical performance, especially in a relatively homogeneous group like this (non-obese strong women) but there are associations between these factors, where %BF is inversely related to strength, while fat free mass shows the opposite relationships.  Normative reference values for the CMJ are presented as percentiles. |
| Robitaille et al. [68] | First, investigate associations between self-reported information and physical performance tests and MSKI sustained by Canadian Armed Forces members enrolled on a developmental period 1 basic infantry course. Second, determine the validity of those measures as predictors of MSKI. | A model including IMTP Peak Force, sandbag drag, Estimated VO_2_peak, and self-reported previous history of MSKI predicted 40% of MSKI in a sample of Developmental Period 1 basic infantry course Infantry candidates, and the results were replicated in a validation sample. Measures of absolute muscular strength and cardiorespiratory fitness, together with self-reported previous history of MSKI were found to predict MSKI in two separate infantry cohorts. |
| Rue et al. [76] | Quantify changes in physical performance pre- and post-basic training using the physical fitness tests of 2 km Run, Medicine Ball Throw, and Mid-Thigh Pull for Army-Junior Entry, Army-Senior Entry, and RAF recruits: (1) at the whole-group level, and (2) relative to recruits’ pre-Basic Training physical performance. | At the whole-group level, 2 km Run time improved in all BT courses, medicine ball throw distance improved for both Army-Junior entry (JE) and Army-Senior entry (SE) recruits, whereas IMTP force improved for Army-JE only. Additionally, for all courses, individuals in the lowest physical performance quartile pre-basic training demonstrated greater improvements compared to those in the highest quartile, for the 2 km Run and IMTP. |
| Scott et al. [59] | First, determine how changes in Sparta™ Jump Scans compare to changes in various physical fitness measures of US Air Force Airmen during an 8-week SW preparatory course. Second, evaluate if Sparta™ Jump Scans are correlated to physical fitness measures utilized within the Special Warfare Training Wing (SWTW) pipeline. | The Sparta™ Score does not reflect change in a USAF Airman’s physical fitness during an 8-week Special Warfare preparatory course (SW PREP). The Sparta™ Score was not correlated to comprehensive physical fitness as captured by current fitness tests within the USAF Special Warfare training pipeline. The vertical jump force-plate scans assessed in this large cohort study produced scores that did not correlate to the physical fitness of Air Force Special Warfare trainees. In fact, the scores only accounted for ≤3% of the variance observed in the physical fitness tests and are not a valid proxy for commonly used fitness metrics. |
| Šimenko et al. [73] | Explore some additional functional tests for baseline fitness and to evaluate how they are correlated with the current Army physical fitness test scoring system. The newly suggested battery includes the following tests: countermovement jump (CMJ) testing, stork balance test, pull-ups, single leg hamstring bridge test, and loaded prone plank test—MANUAL battery. | Functional tests used in the current study measured new qualities of baseline fitness that are not covered by the traditional army physical fitness test (APFT). This is shown by the significant but weak associations of those tests with APFT, but at the same time, increasing APFT score was significantly associated with better performance on the MANUAL tests. |
| Smith et al. [71] | Examine the feasibility and effects of force plate technology to support an individualised training program in reducing MSKI risk (as defined by muscular strength, balance, muscle asymmetry and cardiorespiratory fitness). | A program of individualised physical training was feasible and produced similar physical, occupational and Sparta Science performances to that of standard, group-designed, physical training in military personnel. The results of the current study indicate that force plate technology may have utility in supporting more individualised physical training programs to improve occupational performance and/or reduce injury risk profiles. |
| Solberg et al. [64] | Evaluate the effects of 2 different periodization programs (Linear Periodisation [LP] and Non-Linear Periodisation [NLP]), combined with Block Periodisation, on changes in physical performance and fitness, as well as to evaluate the practical application in an SOF unit. | Results support the benefits of combined periodized programming and individualized training sessions among SOF operators with initially good fitness levels. Largest effects were observed in the first phase with the LP. However, subsequent NLP additionally increased anaerobic and jumping capacity, possibly due to more frequent muscular endurance and power training. Because of frequent interruptions, the LP tended to be more difficult to follow than the NLP and is suggested when continuity is possible, whereas an NLP is recommended for maintenance and when standing on national preparedness. |
| Thompson et al. [28] | Develop and empirically validate an operationally relevant holistic assessment paradigm (markers of performance readiness) to advance the standard of practice by which practitioners test, train, and care for military personnel over a series of 7 events over 4 days. | This study identified psychophysiological, cognitive, and musculoskeletal markers of operational readiness and resilience. Practitioners should leverage daily heart rate variability (HRV) measurements, cognitive assessment apps, and periodic CMJs to understand readiness for and response to training loads. |
| Vikmoen et al. [65] | First, to investigate sex differences in the acute effects of an extremely demanding military field exercise on explosive strength and anaerobic performance, body composition and blood biomarkers related to body composition and physical performance. Second, to examine the recovery in both men and women and investigate if there are any sex differences in the recovery process. | The strenuous field exercise led to large reductions in physical performance that did not differ between men and women. However, the recovery of explosive strength in the lower body was faster in woman than in men. Recovery of physical performance was slow, especially for explosive strength in the lower body, that was not recovered in neither men nor woman two weeks after the exercise. |
| Vikmoen et al. [66] | Investigate how different aspects of physical performance are affected by and recovers from a demanding short-term military field exercise. A special focus in this study was to investigate the underlying physiological and cellular mechanisms behind the decrements in physical performance and investigate any sex differences in these changes. | The 10-day military field exercise had larger negative effects on the ability to produce force at high contraction velocities and explosive strength compared with isometric strength, as peak leg extension torque at 240°·s^−1^ and CMJ height were significantly impaired, whereas no impairment was observed in isometric maximum voluntary contraction leg extension torque. Furthermore, the ability to produce force at high contraction velocities was not recovered in the 2-wk recovery period. |
| Vodičar et al. [74] | Examine BMI effects on baseline fitness that was measured by army physical fitness test (APFT) and additional functional performance tests (vertical countermovement jump with and without load, loaded prone plank, single-leg hamstring bridge test and pull-ups). Our secondary goal is to explore if regular strength training modifies the BMI effect on baseline fitness. | The main finding of our study indicates that additional strength training with regular military physical training significantly modifies negative BMI effect on baseline physical characteristics of soldiers in our sample. This means that additional strength training should be advised to overweight soldiers, if they want to improve their baseline fitness. |
| Walters et al. [12] | First, document the acceptability of introducing the IMTP as a measure of functional strength with military personnel undergoing residential hip pain rehabilitation. Second, compare the peak forces generated by this patient group against the updated Army physical employment standards (PES) assessment criteria. Third, assess if the minimum PES required of military personnel has the potential to inform clinical decision making and return to duty criteria within UK Defence Rehabilitation. | Full patient acceptability, adherence to IMTP test procedures and minimal patient burden were demonstrated. Functionally compromised patients with hip pain in this study produced higher mean net peak force values (131 ± 45 kg) than the criterion values used in the Army PES without a significant change in pain scores. The usefulness of employing the current Army PES criterion values to inform clinical decision making remains unclear. |
| Yanovich et al. [81] | Evaluate gender differences in physical fitness before and after a 4-month gender-integrated basic training (BT) course and to determine whether this program effectively narrowed the differences between male and female soldiers in physical fitness parameters. | There was only a small overlap in physical abilities at the beginning of BT, which indicated vast differences in physical fitness between the genders. Females significantly improved their scores in the Israeli Defence Forces physical fitness test (IDF-PT) and laboratory aerobic tests, whereas males significantly improved only in the IDF-PT. After basic training, gender differences narrowed by approximately 4% in all tests except upper body strength. Although fitness improvement after BT was marginally higher in females than males, resulting in a slight narrowing of the gender differences, a significant gender gap in physical fitness still exists after BT. |
| Zifchock et al. [60] | Monitor changes in skeletal muscle mass, upper body power (ballistic push-up), and lower body power (CMJ) in military personnel during a military training exercise at moderate altitude over ∼4 weeks in 2 contrasting environmental conditions (winter versus moderate temperature). | Explosive movements in the lower and upper body are negatively affected by extended military training, seemingly independent of environmental training conditions (i.e., snow) or temperature. Furthermore, no acute effects of altitude on physical performance were detected. Although participants lost body mass because of the training exercise, only a weak relationship was observed between changes in skeletal muscle mass and countermovement jump peak power, with no other associations between skeletal muscle mass and performance. |
