# Supplementary material for: The Use of Force Plate Technology to Measure Force Production Characteristics in Military Personnel: A Scoping Review of Methodological Reporting Practices
Source: Sports Med Open. 2025 Nov 21;11:140. doi: 10.1186/s40798-025-00942-6 (PMC12638576; doi:10.1186/s40798-025-00942-6)
Supplement: Supplementary file 4 — Supplementary Material 4. [file 40798_2025_942_MOESM4_ESM.docx]

**Supplementary File 4**

**Table S4: Results from the Isometric Mid-Thigh Pull**

| **First Author** | **Metrics used** | **Results** |
| --- | --- | --- |
| Chassé et al [77] | Absolute peak force (N) | **All:** 1570±522 (range,744-2669)  **Women:** 1365±463 (range, 810-1985) |
| Doyle et al. [69] | Absolute peak force (N)  Relative peak force (N·kg^-1^)  Likely scaled relative peak force with units reflecting kg.kg | Start of selection course:  **Uninjured:** 3399 ± 371  **Preventable Knee injury:** 3146 ± 307  Those who completed the selection course:  **Uninjured**: pre-selection, 3408 ± 363; post: 2460 ± 290  **Lower-limb injury:** pre, 3449 ± 547; post, 2498 ± 257  **Knee injury:** pre, 3226 ± 231; post, 2429 ± 255  Start of selection course:  **Uninjured:** 3.91 ± 0.44  **Preventable Knee injury:** 3.77 ± 0.57  Those who completed the selection course:  **Uninjured**: pre, 3.81 ± 0.43; post, 3.37 ± 0.41  **Lower-limb injury:** pre, 4.05 ± 0.45; post, 3.59 ± 0.45  **Knee injury:** pre, 3.75 ± 0.56; post, 3.39 ± 0.41 |
| Lovalekar et al. [53] | Relative peak force (N·kg^-1^)  Time to peak force (s),  RFD 0-100ms (N·s^-1^).  Limb asymmetries (%) | **Lower Limb MSKI:** 29.68 ± 6.37  **Not injured:** 32.62 ± 5.51  **Lower Limb MSKI:** 3.47± 1.46  **Not injured:** 3.36 ± 1.43  **Lower Limb MSKI:** 1,819.05 ± 1,413.15  **Not injured:** 2,341.66 ± 1,779.87  **Lower Limb MSKI:** 14.78 ± 11.46  **Not injured:** 15.33 ± 10.65 |
| McFadden et al. [54] | Peak Force (N)  *(does not specify if gross or net value)*  Relative peak force (N·kg^-1^) | **Men:** week 2, 2,587 ± 477; week 11, 2,654 ± 458  **Women:** week 2, 1,734 ± 363; week 11, 1,736 ± 357  **Men:** week 2, 34.6 ± 5.3; week 11, 36.3 ± 4.8  **Women:** week 2, 27.5 ± 4.6; week 11, 28.0 ± 4.8 |
| McFadden et al. [39] | Peak Force (N)  *(does not specify if gross or net value)* | **Total:** week 2, 2170 ± 590; week 11, 2160 ± 588  **Men:** week 2, 2626 ± 415; week 11, 2618 ± 420  **Women:** week 2, 1747 ± 373; week 11, 1736 ± 357 |
| Nevin et al. [75] | Absolute peak force (N)  Relative peak force (N·kg^-1^)  RFD 0-250 m·s^-1^ (N·s^-1^) | **All men:** 2,417 ± 284  **All men:** 28.14 ± 1.99  **All men:** 3,346 ± 868 |
| Peterson et al. [56] | Relative peak force (N·kg^-1^) | Level of performer:  **Men:** Low, 30.56 ± 2.97; high, 38.40 ± 3.62  **Women**: Low, 24.54 ± 2.28; high, 30.78 ± 3.23  **All**: Low, 28.59 ± 3.95; high, 36.08 ± 4.95 |
| Robitaille et al. [78] | Absolute peak force (N) | **Uninjured:** 1776 ± 370 (95%CI, 1692-1861)  **Injured:** 1613 ± 372 (95%CI, 1467-1758) |
| Rue et al. [76] | Peak force (N)  *(does not specify if gross or net value)* | **Army Junior Entry:** pre-BT, 1200 ± 400; week 49, 1300 ± 365  **Army Senior Entry:** pre-BT, 1405 ± 468; week14, 1369 ± 400  **RAF:** pre-BT, 1340±343; week 10, 1342 ± 343 |
| Walters et al. [12] | Net peak force (N)  Relative net peak force (N·kg^-1^)  Peak force (Kg) | **All:** 1281 ± 440  **All:** 17 ± 45  **All:** 131 ± 45 |

**Table S5:** Results from the Countermovement Jump

| **First Author** | **Metrics used** | **Raw data presented** |
| --- | --- | --- |
| Angelviet et al. [61] | - Peak Power (Watts)   They do not specify which phase of the jump they are calculating peak power (i.e., propulsive or braking phase) | No raw CMJ data reported  Peak power was correlated with performance during the evacuation test (r = -0.56). |
| Barrett et al. [47] | SPARTA Outputs:   - Load, - Explode, - Drive,   SPARTA composite scores:   - MSKI Health score - SPARTA™ score | **Load:** graduate, 43.0 (8.12); non-graduate, 41.8 (7.87)  **Explode:** graduate, 38.8 (7.37); non-graduate, 37.4 (6.75)  **Drive:** graduate, 53.4 (9.27); non-graduate, 52.1 (9.94)  **MSK Health:** graduate, 59.4 (4.71); non-graduate, 58.5 (5.74)  **SPARTA score:** graduate, 78.2 (4.20); non-graduate, 76.8 (3.25) |
| Bird et al. [48] | Jump height (cm)  Modified reactive strength index  Braking RFD (N.s^-1^),  Average braking force (N)  Average relative braking force (%BW)  Average propulsion force (N)  Average relative propulsion force (%BW)  Braking net impulse (N.s)  Propulsive net impulse (N.s)  Peak relative propulsive power (W·kg^-1^)  Peak propulsive power (W)  Braking phase duration (s)  Propulsive phase duration (s)  Time to take off (s). | **Men:** 40 ± 7; MSKI, 40 ± 7; no MSKI, 40 ± 7  **Women:** 29 ± 5; MSKI, 28 ± 5; no MSKI, 29 ± 5  **Men:** 0.38 ± 0.09; MSKI, 0.36 ± 0.08; no MSKI, 0.38 ± 0.09  **Women:** 0.27 ± 0.07; MSKI, 0.26 ± 0.06; no MSKI, 0.27 ± 0.07  **Men:** 3,696 ± 1,808; MSKI, 3,349 ± 1,675, no MSKI, 3,782 ± 1,832  **Women:** 2,399 ± 1,205; MSKI, 2,180 ± 1,153; no MSKI, 2,524 ± 1,223  **Men:** 1,226 ± 203 MSKI, 1,235 ± 201; no MSKI, 1,190 ± 207  **Women:** 943 ± 143; MSKI, 907 ± 128; no MSKI, 963 ± 147  **Men:** 155.6 ± 19.3; MSKI, 152.7 ± 19.6; no MSKI 156.4 ± 19.1  **Women:** 149.3 ± 17.3; MSKI 147.7 ± 18.5; no MSKI, 150.2 ± 16.6  **Men:** 1,396 ± 206; MSKI, 1,364 ± 198; no MSKI, 1,404 ± 208  **Women:** 1,026 ± 139; MSKI, 995 ± 125; no MSKI, 1,044 ± 144  **Men:** 177.1 ± 15.7; MSKI, 174.9 ± 13.0; no MSKI, 177.6 ± 16.3  **Women:** 162.2 ± 12.0; MSKI, 161.6 ± 12.1; no MSKI, 162.6 ± 11.9  **Men:** 111 ± 21; MSKI, 109 ± 21; no MSKI, 112 ± 21  **Women:** 81 ± 15; MSKI, 76 ± 14; no MSKI, 83 ± 15  **Men:** 226 ± 33; MSKI, 222 ± 33; no MSKI, 227 ± 33  **Women:** 153 ± 22; MSKI, 147 ± 21; no MSKI, 157 ± 22  **Men:** 54 ± 8; MSKI, 53 ± 7; no MSKI, 54 ± 8  **Women:** 43 ± 6; MSKI, 43 ± 6; no MSKI, 44 ± 6  **Men:** 4,311 ± 820; MSKI, 4,215 ± 789; no MSKI, 4,335 ± 827  **Women:** 2,797 ± 495; MSKI, 2,699 ± 491; no MSKI, 2,853 ± 492  **Men:** 0.28 ± 0.07; MSKI, 0.29 ± 0.08; no MSKI, 0.27 ± 0.07  **Women:** 0.28 ± 0.06; MSKI, 0.28 ± 0.07; no MSKI, 0.28 ± 0.06  **Men:** 0.38 ± 0.05; MSKI, 0.39 ± 0.05; no MSKI, 0.38 ± 0.06  **Women:** 0.40 ± 0.06; MSKI, 0.40 ± 0.05; no MSKI, 0.40 ± 0.06  **Men:** 1.10 ± 0.14; MSKI, 1.11 ± 0.14; no MSKI, 1.09 ± 0.14  **Women:** 1.10 ± 0.13; MSKI, 1.10 ± 0.14; no MSKI, 1.10 ± 0.12 |
| Bird et al. [49] | SPARTA composite scores   - MSKI Health score - SPARTA™ score | **MSKI:** 55.52 ± 5.31  **No MSKI:** 56.73 ± 4.94  **MSKI:** 78.10 ± 3.59  **No MSKI:** 78.50 ± 3.86 |
| Conkright et al. [50] | Jump height (cm)  Peak force prior to take-off (N)  They do not specify which phase of the jump they are calculating force prior to take off (i.e., propulsive or braking phase) | Day 1, baseline (also collected data on day 2, 3 and 4)  **Men:** pre, 26.9 ± 5.3; mid, 24.4 ± 6.7; post, 25.9 ± 7.6  **Women:** pre, 21.0 ± 4.6; mid, 19.2 ± 4.6; post, 18.2 ± 5.2  **Men:** pre, 2,102 ± 439; mid, 2,223 ± 437; post, 2,288 ± 467  **Women:** pre, 1,519 ± 240; mid, 1,642 ± 228; post, 1,661 ± 197 |
| Doyle et al. [69] | Jump height (cm)  Flight time (ms)  Peak Absolute Force prior to take-off  They do not specify which phase of the jump they are calculating force prior to take off (i.e., propulsive or braking phase)  Peak relative force production (N·kg^-1^)  They do not specify which phase of the jump they are calculating relative force (i.e., propulsive or braking phase) | Start of selection course:  **Uninjured:** 41.24 ± 6.80  **Preventable Knee injury:** 39.08 ± 8.41  Those who completed the selection course:  **Uninjured**: pre-selection, 40.60 ± 6.91; post, 34.30 ± 8.36  **Lower-limb injury:** pre, 44.00 ± 11.70; post, 32.70 ± 3.14  **Knee injury:** pre, 40.50 ± 9.18; post, 31.70 ± 10.20  Start of selection course:  **Uninjured:** 509 ± 35.95  **Preventable Knee injury:** 489 ± 61.48  Those who completed the selection course:  **Uninjured**: pre, 505 ± 38.9; post, 401 ± 72.1  **Lower-limb injury:** pre, 495 ± 27.1; post, 448 ± 30.3  **Knee injury:** pre, 499 ± 67.3; post, 426 ± 56.6  Start of selection course:  **Uninjured:** 1908 ± 245  **Preventable Knee injury:** 1929 ± 253  Those who completed the selection course:  **Uninjured**: pre, 1969 ± 250; post, 1794 ± 255  **Lower-limb injury:** pre, 2018 ± 311; post, 1598 ± 213  **Knee injury:** pre, 2022 ± 204; post, 1757 ± 182  Start of selection course:  **Uninjured:** 2.18 ± 0.15  **Preventable Knee injury:** 2.28 ± 0.25  Those who completed the selection course:  **Uninjured**: pre, 2.19 ± 0.16; post, 2.45 ± 0.36  **Lower-limb injury:** pre, 2.37 ± 0.34; post, 2.29 ± 0.27  **Knee injury:** pre, 2.34 ± 0.27; post, 2.53 ± 0.463 |
| Hamarsland et al. [62] | Jump height (cm) | **Baseline:** 38.8 ± 4.2  **Pre-hell week:** 37.7 ± 4.4  **0 hours:** 27.8 ± 7.3  **24 hours:** 29.1 ± 6.2  **72 hours:** 29.0 ± 5.6  **1-week:** 29.4 ± 4.3  **2-weeks:** 32.9 ± 4.1a |
| Hando et al. [51] | SPARTA Outputs:   - Load, - Explode, - Drive   SPARTA composite scores:   - MSKI Health score - SPARTA™ score   Jump height (inch),  Jump Height (converted to cm) | **Load:** All, 48.3 ± 9.1; no MSKI, 48.3 ± 8.7; MSKI, 48.3 ± 9.6  **Explode:** All, 43.5 ± 7.7; no MSKI, 43.6 ± 7.6; MSKI, 43.5 ± 8.0  **Drive:** All, 52.7 ± 8.1; no MSKI, 52.5 ± 7.9; MSKI, 53.0 ± 8.4  **MSK Health:** All, 57.4 ± 5.0; no MSKI, 57.6 ± 5.0; MSKI, 57.1 ± 5.0  **SPARTA Score:** All, 81.0 ± 3.8; no MSKI, 81.1 ± 3.7; MSKI, 80.9 ± 3.8  **Jump height:** All, 16.5 ± 2.4; no MSKI, 16.4 ± 2.3; MSKI, 16.5 ± 2.4  **Jump Height:** All, 41.9 ± 6.01; no MSKI, 41.7 ± 5.8; MSKI, 41.9 ± 6.01 |
| Karatrantou et al. [80] | Jump height (cm) | Whole body vibration (WBV) comparing high (HF) or low frequency (LF)  **HF-WBV:** pre-training, 38.15 ± 10.14; post-training, 38.71 ± 8.55  **LF-WBV:** pre, 37.31 ± 4.71; post, 39.03 ± 8.42  **Control:** pre, 36.88 ± 5.77; post, 37.96 ± 5.63 |
| Kozinc et al. [72] | Jump height (cm)  Average power (W)  They do not specify which phase of the jump they are calculating average power (i.e., propulsive or braking phase)  Average force (N)  They do not specify which phase of the jump they are calculating average force (i.e., propulsive or braking phase)  Push off force impulse (Ns)  *Push-off force impulse represents the positive force impulse, without the braking phase portion.*    Positive force impulse (Ns)  *The positive force impulse was calculated from the interval starting at the end of the unweighing phase and ending with take-off.* | **Intervention:** unloaded (pre), 28.2 ± 3.8; unloaded (post), 29.5 ± 3.3: loaded (pre), 25.3 ± 3.7; loaded (post), 27.1 ± 6.1  **Control:** unloaded (pre), 26.7 ± 5.6; unloaded (post), 26.8 ± 6.2:  loaded (pre), 23.8 ± 5.5; loaded (post), 24.7 ± 7.5  **Intervention:** unloaded (pre), 2252.1 ± 369.2; unloaded (post), 2289.2 ± 360.0: loaded (pre), 2225.3 ± 354.0; loaded (post), 2296.9 ± 399.1  **Control:** unloaded (pre), 2198.6 ± 466.1; unloaded (post), 2190.3 ± 551.5: loaded (pre), 2150.0 ± 475.2; loaded (post), 2146.9 ± 409.6  **Intervention:** unloaded (pre), 1697.1 ± 251.7; unloaded (post), 1689.4 ± 218.6: loaded (pre), 1746.9 ± 238.3; loaded (post), 1760.3 ± 233.2  **Control:** unloaded (pre), 1690.3 ± 270.9; unloaded (post), 1671.7 ± 251.2: loaded (pre), 1732.0 ± 272.1; loaded (post), 1712.9 ± 227.8  **Intervention:** unloaded (pre), 209.0 ± 29.2; unloaded (post), 214.3 ± 26.7: loaded (pre), 217.9 ± 29.8; loaded (post), 222.1 ± 30.3  **Control:** unloaded (pre), 195.8 ± 34.2; unloaded (post), 195.4 ± 38.5: loaded (pre), 203.5 ± 38.1; loaded (post), 202.3 ± 37.4  **Intervention:** unloaded (pre), 316.5 ± 46.8; unloaded (post), 313.5 ± 44.9: loaded (pre), 331.9 ± 46.6; loaded (post), 330.4 ± 48.0  **Control:** unloaded (pre), 286.9 ± 52.9; unloaded (post), 281.1 ± 51.9: loaded (pre), 307.6 ± 57.7; loaded (post), 296.8 ± 54.2 |
| Lovalekar et al. [53] | Jump height (cm),  Relative peak power (maximal power during concentric phase: W·kg^-1^),  Concentric impulse (Ns),  Eccentric deceleration duration (ms),  Eccentric deceleration impulse (Ns).  Limb asymmetries (%)  (using concentric peak force)  Limb asymmetries (%)  (using concentric impulse). | Multiple group comparaisons are provided. Below compares uninjured and lower extremity MSKI during Marine Corps recruit training only  **Lower Limb MSKI:** 25.16 ± 7.60  **Not injured:** 29.98 ± 7.47  **Lower Limb MSKI:** 41.69 ± 7.70  **Not injured:** 46.75 ± 8.02  **Lower Limb MSKI:** 146.98 ± 41.91  **Not injured:** 173.06 ± 38.85  **Lower Limb MSKI:** 162.04 ± 42.15  **Not injured:** 176.30 ± 52.73  **Lower Limb MSKI:** 68.42 ± 23.32  **Not injured:** 77.81 ± 23.86  **Lower Limb MSKI:** 9.53 ± 7.32  **Not injured:** 7.70 ± 6.23  **Lower Limb MSKI:** 7.89 ± 6.14  **Not injured:** 7.05 ± 5.91 |
| McFadden et al. [54] | Peak power (W)  *(does not specify absolute or net peak power)*  They do not specify which phase of the jump they are calculating average power (i.e., propulsive or braking phase)  Relative peak power (W·kg^-1^)  They do not specify which phase of the jump they are calculating average power (i.e., propulsive or braking phase) | **Men:** week 2, 3,699 ± 624; week 11, 3,290 ± 533  **Women:** week 2, 2,329 ± 372; week 11, 2,201 ± 342  **Men:** week 2, 49.9 ± 6.2; week 11, 44.7 ± 5.2  **Women:** week 2, 37.6 ± 4.9; week 11, 35.7 ± 4.2 |
| McFadden et al. [39] | Peak power (W)  They do not specify which phase of the jump they are calculating peak power (i.e., propulsive or braking phase) | **Men:** week 2, 3658 ± 592; week 11, 3300 ± 585  **Women:** week 2, 2358 ± 370; week 11, 2201 ± 342  **Total:** week 2, 2984 ± 814; week 11, 2730 ± 726 |
| Merrigan et al. [46] | Jump height (cm)  Modified reactive strength index  Concentric peak power (W)  Concentric mean power (W)  Eccentric peak force (N)  Braking duration (s)  Eccentric mean power (W)  Eccentric peak velocity (m.s^-1^)  CMJ depth (cm) | **Men:** 37.66 ± 5.62  **Women:** 23.19 ± 3.73  **All:** PVC pipe, 28.1 ± 1.3: 10 kg vest, 26.0 ± 1.3; 20 kg barbell, 21.0 ± 1.3  **Men:** 0.46 ± 0.08  **Women:** 0.31 ± 0.08  **All:** pipe, 0.36 ± 0.02: 10 kg, 0.33 ± 0.01; 20 kg, 0.24 ± 0.02  **Men:** 53.62 ± 7.34  **Women:** 38.04 ± 4.55  **All:** pipe, 41.3 ± 1.5: 10 kg, 43.9 ± 1.5; 20 kg, 35.2 ± 1.5  **Men:** 28.54 ± 4.45  **Women:** 20.39 ± 3.41  **All:** pipe, 22.9 ± 0.8: 10 kg, 21.6 ± 0.8; 20 kg, 18.2 ± 0.8  **Men:** 21.94 ± 3.01  **Women:** 20.28 ± 2.76  **All:** pipe, 20.0 ± 0.4: 10 kg, 19.7 ± 0.4; 20 kg, 17.8 ± 0.4  **Men:** 0.36 ± 0.06  **Women:** 0.35 ± 0.08  **All:** pipe, 0.367 ± 0.009: 10 kg, 0.379 ± 0.009; 20 kg, 0.413 ± 0.009  **Men:** 29 18 ± 6.39  **Women:** 5.80 ± 0.84  **All:** pipe, 6.09 ± 0.11: 10 kg, 6.08 ± 0.11; 20 kg, 5.63 ± 0.11  **Men:** −1.23 ± 0.21  **Women:** −1.11 ± 0.18  **All:** pipe, −1.16 ± 0.02: 10 kg, −1.13 ± 0.02; 20 kg, −1.06 ± 0.02  **Men:** −36.28 ± 7.36  **Women:** −30.31 ± 4.53  **All:** pipe, −32.7 ± 1.1: 10 kg, −33.3 ± 1.1; 20 kg, −34.2 ± 1.1 |
| Øfsteng et al. [63] | Jump height (cm) | The authors do not specify CMJ values within the text.  Visual inspection suggests a mean jump height of 38cm at baseline. At 10 days post military exercise this value drops to ~32cm (~15% decrease) and stays reduced at ~32.5cm after 7-days of recovery. |
| Peterson et al. [56] | Concentric relative peak power (W·kg^-1^). | **Men:** Low performers, 46.9 ± 6.1; high performers, 52.5 ± 5.8  **Women**: Low performers, 36.0 ± 4.6; high performers, 39.6 ± 4.3  **All**: Low performers, 43.3 ± 7.6; high performers, 48.6 ± 8.0 |
| Pihlainen et al. [79] | Jump height (cm) | **Men**: unloaded, 38 ± 6; loaded pre-test, 28.5 ± 5.1, loaded post-test, 27.0 ± 5.0 |
| Potter et al. [58] | Jump height (cm)  Peak power (W)  They do not specify which phase of the jump they are calculating peak power (i.e., propulsive or braking phase)  Relative peak power (W·kg^-1^)  They do not specify which phase of the jump they are calculating relative peak power (i.e., propulsive or braking phase) | **Women**: mean, 22 ± 7.1; Percentiles 5^th^, 12.5cm; 10^th^, 14.5cm; 25^th^, 17.4cm; 50^th^, 21.6cm; 75^th^, 26cm; 90^th^, 31.8cm; 95^th^, 35.6cm.  **Women:** Mean, 2575 ± 565.2; Percentiles 5^th^, 1716.4W; 10^th^, 1898.9W; 25^th^, 2184.3W; 50^th^, 2510.2W; 75^th^, 2933.2W; 90^th^, 3327.4W; 95^th^, 3606.5W.  **Women**: Mean, 37.4 ± 7.3; Percentiles 5^th^, 26.6; 10^th^, 29.2; 25^th^, 32.3; 50^th^, 36.5; 75^th^, 41.8; 90^th^, 47.7; 95^th^, 51.1. |
| Scott et al. [59] | SPARTA Outputs   - Load - Explode - Drive - vertical jump height (cm)   SPARTA composite scores   - SPARTA™ score | **Load**: pre, 48.7 ± 3.7; 8-weeks post, 47.5 ± 7.7  **Explode:** pre, 43.8 ± 7.5; 8-weeks post 42.3 ± 7.1  **Drive:** pre, 52.5 ± 8; 8-weeks post, 50 ± 7.8  **Jump height:** pre, 41.9 ± 5.8; 8 weeks post, 39.1  **Sparta Score:** pre, 81.2 ± 3.7; 8 weeks post, 80.3 ± 3.4 |
| Šimenko et al. [73] | Jump height (cm) | **CMJ (unloaded):** mean, 27.9 ± 4.2  **CMJ (unloaded) percentiles:** 5^th^, 21.9; 10^th^, 22.4; 25^th^, 25.4; 50^th^, 27.5; 75^th^, 30.3; 90^th^, 34.3; 95^th^, 36  **CMJ (loaded):** mean, 25±4.3  **CMJ (loaded) percentiles:** 5^th^, 19.1; 10^th^, 20.6; 25^th^, 22.4; 50^th^, 24.9; 75^th^, 27.3; 90^th^, 30.1; 95^th^, 32.1 |
| Smith et al. [71] | SPARTA Outputs   - Load, - Explode, - Drive - vertical jump height (cm)   SPARTA composite scores   - MSKI risk score - SPARTA™ score | **CMJ Load**: Control baseline, 45.8 ± 10.3; 6-weeks post, 48.5 ± 10.9 \| Experiment group baseline, 40.8 ± 5.3; 6-weeks post, 42.7 ± 2.4  **CMJ Explode:** Control baseline, 47.8 ± 12.6; 6-weeks post, 49 ± 13.6 \| Experiment group baseline, 39.5 ± 4.9; 6-weeks post, 43.7 ± 6.5  **CMJ Drive:** Control baseline, 44.1 ± 12.4; 6-weeks post, 36.8 ± 13.5 \| Experiment group baseline, 51.2 ± 11; 6-weeks post, 44.1 ± 9.6  **CMJ Jump height:** Control baseline, 37.3 ± 10; 6-weeks post, 33.6 ± 8.8 \| Experiment group baseline, 35 ± 8.2; 6-weeks post, 34.4 ± 7.7  **CMJ Injury risk score:** Control baseline, 1.78 ± 1.56; 6-weeks post, 1.89 ± 1.05 \| Experiment group baseline, 1.50 ± 0.93; 6-weeks post, 1.25 ± 0.71  **CMJ Sparta score:** Control baseline,78.8 ± 4.9; 6-weeks post, 76.7 ± 5.7 \| Experiment group baseline, 78.1 ± 3.1; 6-weeks post, 79.3 ± 1.1 |
| Solberg et al. [64] | Jump height (cm) | **All:** baseline, 41.4 ± 3.1  No other raw data presented only trivial changes at **6 month**, -1 ± 5 (95%CI = 2) and **12 months**, 2 ± 5 (95%CI = 3) |
| Thompson et al. [28] | Mean modified RSI  Mean vertical jump height (cm)  Mean Scaled Power | Raw values not presented. |
| Vikmoen et al. [65] | Jump height (cm)  Peak power (W)  They do not specify which phase of the jump they are calculating peak power (i.e., propulsive or braking phase) | **Men:** baseline, 38.7 ± 4.1; post, 31.2 ± 4.1; 24 hours post, 31.3 ± 3.9; 72 hours post, 29.4 ± 3.9; 1-week post, 30.0 ± 4.0; 2-week post, 32.0 ± 4.0.  **Women:** baseline 29.0 ± 3.6; post, 23.5 ± 3.8; 24-hours post, 24.2 ± 3.8; 72 hours post, 24.8 ± 3.4; 1-week post, 24.1 ± 3.7; 2-weeks post, 26.3 ± 3.1.  **Men:** baseline, 3813 ± 511; post, 3144 ± 434; 24-hour post, 3209 ± 441; 72-hours post, 3080 ± 437; 1-week post, 3177 ± 457; 2-weeks, 3409 ± 508.  **Women:** baseline, 2650 ± 384; post, 2265 ± 280; 24-hours post, 2387 ± 357; 72 hours post, 2394 ± 309; 1-week post, 2388 ± 333; 2-weeks post, 2540 ± 310 |
| Vikmoen et al. [66] | Jump height (cm) | **Men**: baseline, 38.0 ± 8.6; 24 hours post, 35.1 ± 9.3; 1-week post, 35.2 ± 8.9; 2-week post, 36.5 ± 9.1  **Women**: baseline, 28.8 ± 4.2; 24-hours post, 27.4 ± 4.1; 1-week post, 26.4 ± 3.8; 2-week post, 27.6 ± 3.6 |
| Vodičar et al. [74] | Jump Height (cm) | **CMJ (unloaded) strength training:** normal BMI, 29.9 ± 4; overweight, 29 ± 4.3; obese, 29.2±4  **CMJ (unloaded) no strength training:** normal BMI, 30.2 ± 4.4; overweight, 26.2 ± 3, obese, 26.4 ± 1.6  **CMJ (loaded) strength training:** normal BMI 24.3 ± 3.7; overweight, 26 ± 3.9; obese, 27.9 ± 4.8  **CMJ (loaded) no strength training:** normal BMI, 26.1 ± 3.7; overweight, 23.8 ± 3.9; obese, 23.9 ±2.2 |
| Yanovich et al. [81] | Relative Force (N.kg^-1^)  They do not specify which phase of the jump they are calculating relative force (i.e., propulsive or braking phase)  Relative Power (W.kg^-1^)  They do not specify which phase of the jump they are calculating relative power (i.e., propulsive or braking phase) | **Men:** Pre, 49.9 ± 12.9; post, 44.6 ± 8.3  **Women:** pre, 46.1 ± 9.2; post, 42.7 ± 7.2  No Relative Power data is presented for CMJ  Despite stating in the methods that they completed a SL CMJ and recorded relative force and relative power for this test, no data is presented |
| Zifchock et al. [60] | Concentric peak power (W)  Jump height (cm) | **All:** Baseline, 4009 ± 902; Mountains, 4002 ± 866  **All:** Baseline, 33.9 ± 5.8; Mountains, 33.6 ± 5.6 |
